# Synergistic effect of grassland plants and beneficial rhizosphere bacteria helps plants cope with overgrazing stress

**DOI:** 10.1186/s12870-025-06638-z

**Published:** 2025-05-10

**Authors:** Ting Yuan, Weibo Ren, Jiatao Zhang, Mohsin Mahmood, Zhenyu Jia, Shaohong Zhang, Min Wang, Shuang Liang, Feng Yuan, Yaling Liu

**Affiliations:** 1 Inner Mongolia Key Laboratory of Grassland Ecology and the Candidate State Key Laboratory of Ministry of Science and Technology, Inner Mongolia University, Hohhot, 010010 China; 2 National Center of Pratacultural Technology Innovation, Hohhot, China

**Keywords:** Gene expression, *Leymus chinensis*, Multi-omics analysis, Overgrazing, Plant-growth promoting bacteria, Root exudate

## Abstract

**Background:**

Overgrazing (OG) is an important driver of grassland degradation and productivity decline. Highly effective synergy between plants and rhizosphere growth-promoting rhizobacteria (PGPR) may be a major way for grassland plants to effectively cope with OG stress. There have been few reports providing solid evidence on how this synergy occurs.

**Result:**

This study combined with multi-omics analysis and the interaction effect of specific root exudate with PGPR B68, aiming to reveal the synergistic effect and regulatory mechanism of *L. chinensis* and PGPR under overgrazing stress. The results showed that *Leymus chinensis* plants with OG history can recruit the beneficial *Phyllobacterium* sp. B68 by regulating specific root exudate compounds(such as amino acid L-leucyl-L-alanine and alkaloid cordycepin). These compounds enhanced B68 rhizosphere colonization by promoting B68 chemotaxis and biofilm formation. The pot study experiments indicated that the bacterial isolates used as bio inoculants increased *L. chinensis* growth (mainly including plant height and biomass) by significantly increasing the chlorophyll content, RuBisCO activity, soluble sugar, plant hormones and nutrient content. Metagenomics results show that B68 inoculation significantly altered rhizosphere soil bacterial community composition and function. Additionally, B68 systemically upregulated the expression level of genes involved in plant hormone signaling, nutrient and sugar transporters, nitrogen metabolism, cell division, cell wall modification and photosynthesis to promote plant growth. The above results indicate that the PGPR B68 recruited by the root exudates of *L. chinensis* under OG helps the plant adapt to stress by promoting nutrient uptake and transport, maintaining hormone homeostasis, and enhancing the expression of genes related to plant growth and nutrient metabolism.

**Conclusion:**

This study provides new insights into the positive interactions between grassland plants and rhizosphere bacteria under OG stress, offering valuable knowledge for developing new fertilizers and better management practices for degraded rangeland restoration and sustainable agriculture development.

**Clinical trial number:**

Not applicable.

**Supplementary Information:**

The online version contains supplementary material available at 10.1186/s12870-025-06638-z.

## Introduction

Grasslands play a vital role in maintaining global ecosystem functions and are widely utilized for livestock production [1]. Grassland degradation, often driven by climate change and improper human activities such as overgrazing (OG), poses a significant threat to these ecosystems [1, 2]. Grazing, the most prevalent form of land use globally, strongly influences soil microbial communities. Herbivores impact aboveground and belowground biomass and soil microbial communities through trampling, defoliation, and dung and urine deposition [3]. Reduced aboveground biomass due to grazing can lead to an increased allocation of plant biomass belowground and increased root exudates, further influencing the microbial community [4]. Although moderate grazing can positively impact soil bacterial communities and plant growth by promoting biodiversity and productivity, OG reduces the density and biomass of high-quality grasses, adversely affecting the soil microbial community [2]. However, the effects of plant interactions with rhizosphere microbial communities under OG stress remain unknown.

Plants in terrestrial ecosystems respond dynamically to environmental changes by altering morphological or physiological traits (phenotypic plasticity), which enhances their fitness [5]. Under biotic or abiotic stresses, plants can recruit beneficial soil microbes via root exudates to resist these stresses (i.e., “cry for help” strategy) [6, 7]. This recruitment alters microbial community composition and functions, enriching plant growth-promoting rhizobacteria (PGPR) such as *Phyllobacterium* sp., *Pseudomonas* sp., *Citrobacter* sp., and *Flavisolibacter* sp [8, 9]. These microbes facilitate the acquisition of essential mineral nutrients, promoting mutualistic plant–microbe interactions [10]. Microbes obtain carbon (C) and nitrogen (N) from root exudates, which contain amino acids, sugars, nucleotides, terpenes, phenylpropanoids, and flavonoids [11]. These metabolites enhance nutrient acquisition, regulate beneficial microbes, and contribute to plant growth and development, stress resistance, and environmental adaptation [12].

Through a variety of processes, beneficial microbes greatly improve plant establishment, growth, and development [13]. PGPR, isolated from rhizosphere soils, play a direct role in aiding atmospheric nitrogen fixation, solubilization of complex phosphate, production of siderophores, synthesis of plant hormone, improve soil properties, and enhance plant tolerance, providing fitness advantages to the host plant [14, 15]. Despite incomplete understanding of the PGPR-mediated plant growth mechanisms, several well-characterized PGPR have been useful in agriculture, boosting plant nutrient absorption, growth, yield, stress resistance, and phytohormone modulation [16–18]. PGPR inoculation significantly influences root and leaf development, nutrient uptake, and metabolite regulation [19, 20].

The “bottom-up” regulation by PGPR of plant functional genes involved in plant nutrient uptake and growth is crucial for agricultural productivity. Beneficial microbes regulate genes involved in nutrient absorption, ion transport, and secondary metabolite production, thereby promoting plant growth [21]. For example, PGPR systemically regulate gene transcription related to growth, development, nutrient absorption, and metabolism, particularly auxin responses and nutrient signaling pathways, enhancing nutrient absorption and promoting N and P recycling within plants [22, 23]. Selectively enriched rhizosphere microbes can prolong N bioavailability, produce phytohormones, delay flowering, and downregulate genes triggering flowering to stimulate further plant growth [24]. This indicates that beneficial rhizosphere microbes can modulate plant growth and development by producing hormones and mobilizing nutrients for efficient uptake.

Plant–microbe interactions promote plant health, growth, and development. With advancements in omics technologies, the synergistic regulatory mechanisms of plant–microbe interactions have been increasingly elucidated. Under feeding stress from small herbivores, plants actively regulate their root exudates to shape rhizosphere microbial communities, triggering immune responses in aboveground organs and enhancing resistance to herbivores [25]. In addition to increasing hormone (indole-3-acetic acid [IAA]) synthesis and nutrient availability, rhizosphere bacteria can improve plant regeneration and tolerance following herbivore feeding [26]. Recent studies have shown that grazing disturbance reshapes plant rhizosphere microbial communities, with feedback effects on plant growth [27, 28].

In our previous study, *Leymus chinensis* was found to recruit beneficial microbes by altering root metabolites, which enhanced plant growth and resistance to grazing stress, thereby improving environmental adaptability [29]. Under long-term OG stress, *L. chinensis* might regulate its synergistic effects involving rhizosphere bacteria via root metabolites [30]. However, there is limited evidence on the mechanisms of this synergy under OG stress. Key issues include identifying the beneficial bacteria enriched after OG, understanding how these bacteria are recruited by root exudates, and determining how they help plants to adapt to OG stress.

**Fig. 1:**
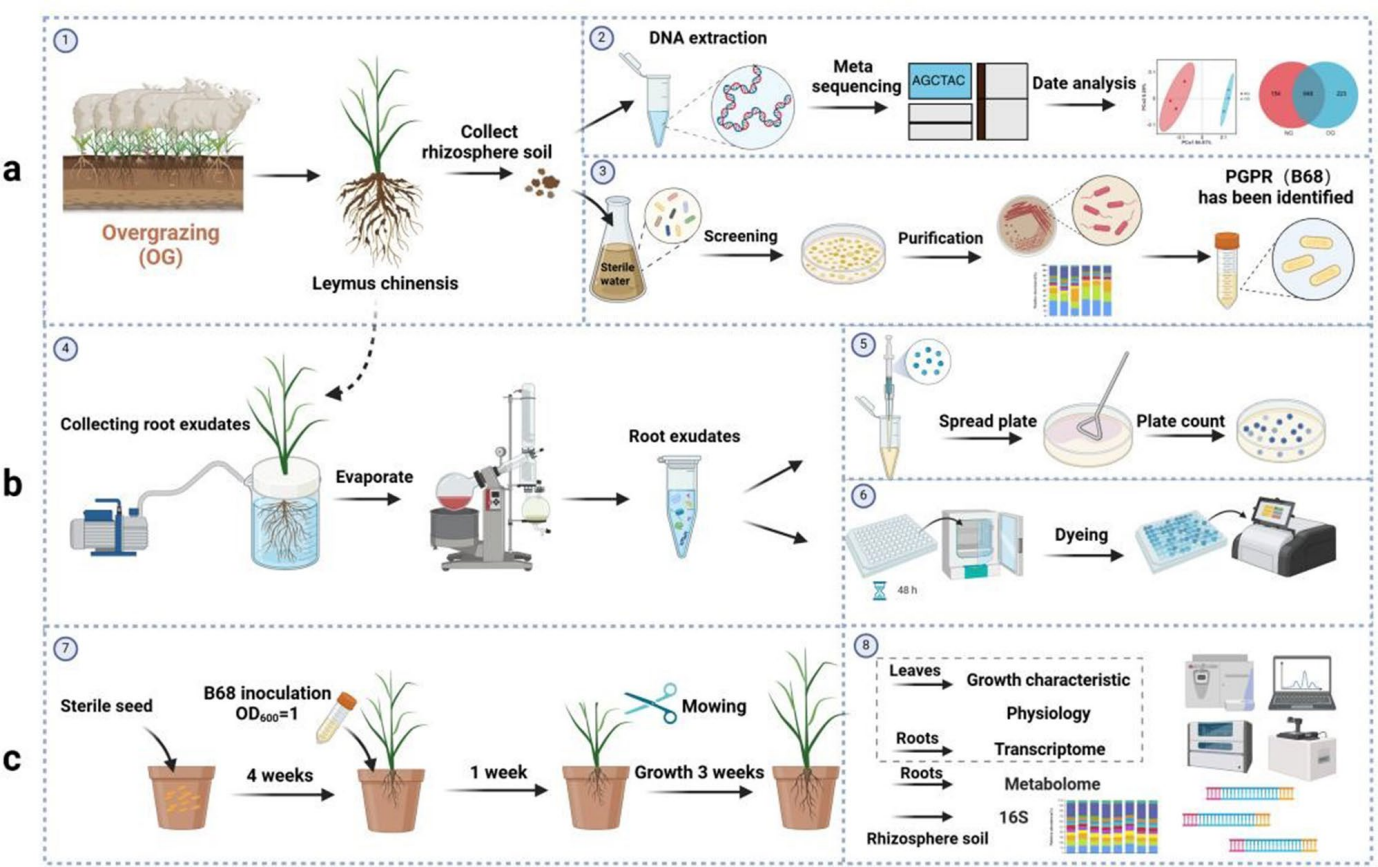
Schematic representation of the experimental design. (1) *L. chinensis* rhizosphere soil was collected from areas with long-term overgrazing (OG) or no grazing (NG). (2) The rhizosphere soil was subjected to metagenome sequencing. (3) *Phyllobacterium* sp. B68 was isolated and tested for growth-promoting properties. (4) *L. chinensis* root exudates were collected. PGPR recruitment by specific root exudate compounds was assessed based on B68 (5) chemotaxis and (6) biofilm formation. (7) B68 was inoculated on *L. chinensis* seedlings, which underwent mowing treatment to simulate grazing. (8) Finally, the effects of B68 inoculation on the rhizosphere microbial community, *L. chinensis* growth characteristics and physiological response, and the growth-promoting mechanism (based on transcriptomics and metabolomics) were analyzed

In our present study, we engrossed on *L. chinensis*, the dominant species of typical grassland in Inner Mongolia, combined with multi-omics study (involving metagenomics, transcriptomics, and metabolomics), explored the synergistic and regulatory mechanism between grassland plants and PGPR under OG stress. The objectives were (a) to analyze whether OG alters root exudates to recruit beneficial microbes; to explore the effects of OG on the bacterial community, functional genes, and key taxa in *L. chinensis* rhizosphere soil; and to screen, isolate, and purify key PGPR and study their growth-promoting properties; (b) to test whether specific *L. chinensis* root exudate compounds recruit PGPR by promoting chemotaxis and biofilm formation; and (c) to determine the *L. chinensis* phenotype and physiological response (including growth promotion) after PGPR inoculation and to analyze whether PGPR can successfully colonize the *L. chinensis* rhizosphere and the molecular mechanism underlying *L. chinensis* growth promotion after PGPR inoculation (Fig. 1). Our findings clarify the synergistic effects of plant–microbe interactions in grazing grassland ecosystems and provide scientific support for studying the response mechanisms of grassland plants under OG stress, optimizing grassland management, and rapidly restoring and improving grassland productivity.

## Materials and methods

### Study site and sampling

The study site was located near the Grassland Ecosystem Research Station in Inner Mongolia, China (43°38′30″N, 116°42′20″E, and 1250 m a.s.l.). The region has a semi-arid climate, with a mean annual precipitation of 340 mm, 60–80% of which falls from May to September [31]. The grasses *L. chinensis*, *Agropyron cristatum*, and *Stipa grandis* are the dominant plant species and widely distributed in the Eurasian steppe [32]. The study site is separated into two areas by a pasture fence: (1) no grazing (NG) area (600 × 400 m; in this enclosed grazing-exclusion area, long-term ecological observation studies have been conducted since 1983) and (2) overgrazed (OG) area (600 × 100 m; this area has been continuously overgrazed [~ 3 sheep/ha] for > 50 years from June to October every year). The NG and OG areas are continuously distributed on the same upper basalt platform, helping to control soil heterogeneity [33]. Sampling was carried out at the end of July 2022, with each of the two areas divided into three plots. *L. chinensis* plants and *L. chinensis* rhizosphere soil (for rhizosphere bacterial isolation and microbial community analysis) were collected from each plot. *L. chinensis* rhizosphere soil and root exudate collection are described in detail in the Supplementary Methods.

### Bacterial isolation, identification and testing of growth-promoting properties

The rhizosphere-promoting bacteria were isolated from *L. chinensis* rhizosphere soils in the OG group using Tryptic Soy Broth (TSB), Reasoner’s 2 A Agar (R2A) and Luria-Bertani Broth (LB) media. Briefly, rhizosphere soil samples were suspended in phosphate-buffered saline, subjected to gradient dilution (10^− 3^, 10^− 4^, 10^− 5^, and 10^− 6^), and incubated on TSB, R2A, and LB media for 5–7 days at 30 °C for bacterial isolation. Single colonies were purified by triple serial colony isolation onto their respective media using a sterile inoculation loop and different isolates were selected based on morphological characteristics and colony size. Strains were identified by 16 S rRNA sequencing and their growth-promoting properties (IAA production, inorganic phosphate-solubilizing activity, and nitrogenase activity) were assessed in detail in the Supplementary Methods. Their 16 S rRNA genes were amplified with primers F27 (5’-AGTTTGATCMTGGCTCAG-3’) and R1492 (5’-GGTTACCTTGTTACGACTT-3’). Sequencing results were analyzed by NCBI BLAST, and the phylogenetic tree was established by MEGA 11 software. Based on the metagenomic sequencing results (some PGPR, such as Phyllobacterium, were present only under OG), we isolated it from the overgrazed *L. chinensis* rhizosphere soil and used it as a model strain for subsequent pot experiments to investigate its growth-promoting mechanisms for *L. chinensis*.

### Pot experiments

*L. chinensis* seeds (West Ujumuqin Leymus, provided by Grassland Research Institute, Chinese Academy of Agricultural Sciences) were soaked in 75% ethanol for 2 min and 2% NaClO solution for 10 min and then thoroughly washed. The uniformly sized seeds were grown in a culture dish for 10 days. After growing to the trefoil stage, the seedlings were transplanted into pots (height, 20 cm; diameter, 18 cm) containing approximately 3 kg of field soil, substrate soil, and vermiculite (4:1:1 volume ratio) for 30 days. There were eight seedlings per pot and each replicate contained six pots. The pots were randomly placed in a greenhouse (16/8 h light/dark cycle, 30 °C/18°C (day/night), relative humidity 60–70%), soil moisture was maintained at 60% and their positions were randomly changed every 3 days. B68 cells from LB cultures were centrifuged (8000 g for 5 min) to harvest the cell pellets, which were then washed and resuspended in sterile 0.9% NaCl solution. The B68 suspension was adjusted to approximately 10^8^ CFU mL^− 1^ (OD600 = 1.0). Four-week-old *L. chinensis* plants were inoculated by slowly adding 10 mL B68 suspension (by adding it to the soil around each plant root) or left non-inoculated (control) to the soil around each plant root using a 10-mL sterile syringe. After 1 week, mowing treatment (stubble height, 4 cm) was used to simulate grazing [34]. At 4 weeks after B68 inoculation, plant height, stem diameter, leaf width, leaf length, chlorophyll content, fresh shoot weight, fresh root weight, dry shoot and root weights were measured. The whole roots and leaves of two pots of *L. chinensis* were mixed into one sample with a total of three samples used as biological replicates, and immediately frozen and stored at − 80 °C for physiological indices, transcriptome and metabolome evaluation. Rhizosphere soil was collected for rhizosphere microbiome analysis.

### Determination of phytohormones, nutrient content and enzymatic activity

The bicinchoninic acid (BCA) protein concentration, soluble sugar content, malondialdehyde (MDA) content, and superoxide dismutase (SOD), lipoxygenase (LOX), polyphenol oxidase (PPO), peroxidase (POD), phenylalanine ammonia-lyase (PAL), and catalase (CAT) activities in root or/and leaf samples in the B68-inoculated and non-inoculated groups were measured using corresponding assay kits (Suzhou Keming Biotechnology Co., Ltd., China) [35]. RuBisCO activity, sucrase activity, glucose, total carbon (C) and total phosphorus (P) were measured by Suzhou Keming Biotechnology Co., Ltd. About 0.3 g of dried plants were ground into powder and the total nitrogen (N) and total potassium (K) contents were measured using a graphite digester (S402, Shandong, China) according to Chen et al. (2018) [36]. Liquid chromatography-tandem mass spectrometry (LC–MS/MS) (Rigol L3000, Beijing, China) was used to assess abscisic acid (ABA), jasmonic acid (JA), indole-3-acetic acid (IAA), gibberellin (GA_3_) and salicylic acid (SA) in fresh plants according to Castro-Valdecantos et al., (2021) [37]. The chlorophyll content was determined using a Soil Plant Analysis Development (SPAD)-502 Plus chlorophyll meter (Konica Minolta, Japan).

### In vitro chemotaxis and biofilm formation assays

The effects of seven root exudate compounds on B68 chemotaxis and biofilm formation were assessed using modified capillary assays and biofilm formation assays, respectively. Details are described in the Supplementary Methods.

### Metagenomic sequencing of *L. chinensis* rhizosphere soil

Total DNA extraction, DNA concentration and quality assessment, and sequencing library construction are described in the Supplementary Methods. Metagenomic paired-end sequencing of *L. chinensis* rhizosphere soil in the NG and OG groups was performed using a NovaSeq X Plus sequencer (Illumina) by Gene Denovo Biotechnology Co., Ltd. (Guangzhou, China). FASTP v0.18.0 was used to remove adapter sequences and low-quality reads from the raw reads [38]. Gene Ontology (GO) and Kyoto Encyclopedia of Genes and Genomes (KEGG) analyses were conducted for functional annotation.

### Amplicon sequencing of 16 S rRNA gene of *L. chinensis* rhizosphere soil

To determine whether B68 can successfully colonize and alter the rhizosphere bacterial community, amplicon sequencing was performed. A HiPure Stool DNA Kit (Magen, Guangzhou, China) was used to obtain the total DNA from *L. chinensis* rhizosphere soil in the B68-inoculated and non-inoculated groups. The V3-V4 region of the 16 S rRNA gene was amplified using the specific primers 341 F (5′-CCTACGGGNGGCWGCAG-3′) and 806R (5′-GGACTACHVGGGTATCTAAT-3′) [39]. Cycling conditions consisted of 5 min at 95 °C, 30 cycles of 95 °C for 1 min, 60 °C for 1 min, and 72 °C for 1 min, followed by 7 min at 72 °C. The purified PCR products were sequenced on an Illumina NovaSeq 6000 SP PE250 platform by Gene Denovo Biotechnology Co., Ltd. (Guangzhou, China). Raw reads were assembled and quality-filtered using FASTP v0.18.0. Representative amplicon sequence variants (ASVs) were classified using RDP classifier v2.2 [40].

### Transcriptome sequencing and quantitative PCR analyses

Total RNA was extracted from *L. chinensis* roots and leaves in the B68-inoculated and non-inoculated groups, as detailed in the Supplementary Methods. The expression of each transcript was assessed by comparing each transcript to transcripts per million mapped reads (TPM) using the exon model per kilobase [41], and then the expression of each gene was normalized. The DESeq2 software package was used to identify the differentially expressed genes (DEGs) [42] based on|log2fold change (FC)|>1 and adjusted p-value (p_adj_) < 0.05. Based on the transcriptomic results, several genes related to auxin synthesis and signaling, photosynthesis, nutrient uptake, and metabolism were selected for qRT-PCR validation. The primers are listed in Table S1. The relative expression levels of target genes were calculated with Actin gene, which is an internal control gene, using the 2^–∆∆Ct^ method.

### Metabolomic profiling of *L. chinensis* roots and root exudates

Metabolite profiling was conducted according to Liu et al. (2020) [43]. The details are described in the Supplementary Methods. Ultra-performance liquid chromatography (UPLC)-MS/MS-based broad-spectrum metabolomics analysis was used to analyze *L. chinensis* root exudate changes under OG. UPLC-MS/MS-based broad-spectrum metabolomics analysis was used to determine the differential expressed metabolites (DEMs) in *L. chinensis* roots between the B68-inoculated and non-inoculated groups. DEMs were identified based on *p* < 0.05 and variable importance in the projection (VIP) > 1.

### Statistical analysis

The data are presented as mean ± standard error. Means were compared by one-way analysis of variance (ANOVA) followed by Tukey’s Studentized range (honestly significant difference [HSD]) test in SPSS v26. Root exudate composition and bacterial community composition were compared between two groups using permutational multivariate ANOVA (PERMANOVA; Adonis function, data transformed by Bray–Curtis distance metric, 999 permutations). Correlations between root exudates and rhizosphere bacteria were analyzed using the Hmsic package in R v4.0.2 and plotted using Cytoscape. The KEGG functions of bacterial communities were predicted by PICRUSt software [44]. Figures were created using Adobe Illustrator 2022, Origin 2022, RStudio, and the OmicShare platform (https://www.omicshare.com/).

## Results

### Effects of long term-overgrazing on the rhizosphere bacterial communities

**Fig. 2:**
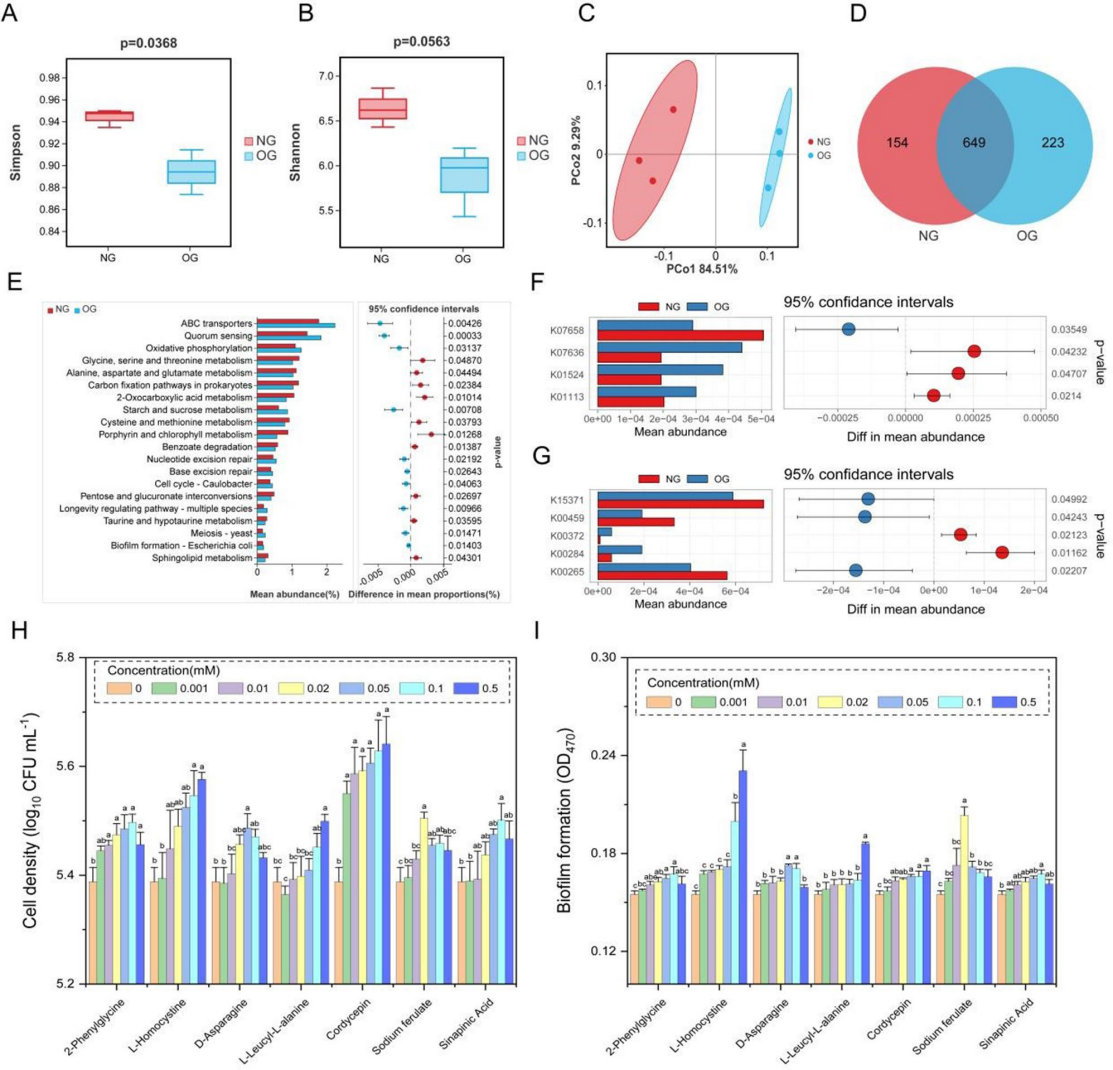
Effects of overgrazing (OG) on rhizosphere bacterial communities. (**A, B**) Simpson and Shannon indices of bacterial communities. (**C**) PCoA of rhizosphere bacterial β-diversity in no grazing (NG) and OG groups. (**D**) Venn diagram of number of bacterial genera. (**E**) Significant differences in mean abundances of KEGG pathways between NG and OG groups. (**F, G**) Differences in functional genes related to N and P cycles. Effects of selected L. chinensis root exudate compounds on B68 (**H**) chemotaxis and (**I**) biofilm formation. Data are shown as mean ± SE (*n* = 3)

There were 394,108,738 raw sequence reads (62,333,908 to 71,118,986 per sample). After quality filtering, the retention rate of clean reads was > 99.6%, and the retention rate of reads with quality score ≥ 30 was > 92% (Table S2). Regarding the bacterial alpha diversity, the Simpson index was significantly decreased in the OG group (*p* < 0.05) (Fig. 2A). Principal component analysis (PCA) revealed clear differences in the rhizosphere bacterial community between the NG and OG groups (Fig. 2C). There were 649 bacterial genera shared by the NG and OG groups and 223 that were only present in the OG group (Fig. 2D). This suggested that OG enriched some bacteria in the rhizosphere microbial community, including PGPR such as Leifsonia [45], Ramlibacter [46], Flavobacterium [47], and Phyllobacterium [48]. KEGG analysis showed that Carbohydrate metabolism (2431), Amino acid metabolism (2358), Metabolism of cofactors and vitamins (2210), and Energy metabolism (1617) had the largest number of unigenes (Fig. S1A). OG significantly enriched Membrane transport, Cell growth and death, and Cellular community – prokaryotes (Fig. S1B).

There were 26 genes detected that were involved in soil microbial phosphorus (P) turnover and P-cycling potential (Table S3). OG enriched genes involved in soil microbial inorganic P solubilization and organic phosphate mineralization (phnG, phnJ, phnF), P transport and uptake (ugpA, ugpB, phnC, ugpE, phnE) (Fig. S1C). In addition, OG rhizosphere soil of *L. chinensis* significantly increased the genes responsible for inorganic phosphorus solubilization (K01524) and alkaline phosphatase (K01113) (Fig. 2F). There were 37 KEGG Orthologues related to the N cycle (Fig. S1D; Table S4), which were involved in N fixation, nitrate reduction, denitrification, ammonification, and N metabolism and significantly increased the genes responsible for glutamate synthase (K00284), methane monooxygenase (K10946) and nitrate reductase (K00372) (Fig. 2G).

### Strain B68 isolation and growth-promoting characteristics

The rhizosphere-promoting bacteria B68 was isolated from the *L. chinensis* rhizosphere soil of overgrazing grassland in Ordos City, Inner Mongolia. Phylogenetic analysis (based on16S rRNA gene sequences) showed B68 is a species of Phyllobacterium, named *Phyllobacterium sp*. B68. This strain is preserved in China General Microbiological Culture Collection Center (CGMCC), No.31,906. A transparent circle was formed around the B68 colony, indicating that it had phosphate-solubilizing ability (Fig. S2A), and the phosphate-solubilizing ring (D)/colony diameter (d) ratio was 1.85 ± 0.16 (Fig. S2A; Table S5). B68 was cultured in LB medium with L-tryptophan (100 mg/L) for 2 days, mixed with Salkowski solution, and incubated in the dark for 30 min. The culture medium turned pink, indicating IAA production (Fig. S2B). B68’s IAA production, inorganic phosphate-solubilizing activity, and nitrogenase activity were 35.49 µg/mL, 87.83 µg/mL, and 183.43 IU/L, respectively (Table S5), indicating that the bacterium had the ability to solve phosphorus, fix nitrogen and produce IAA.

### Effects of *L. chinensis* root exudate compounds on B68 chemotaxis and biofilm formation

There were 144 DEMs (72 upregulated and 72 downregulated) between the NG and OG groups (Fig. S3A, B). KEGG analysis of these DEMs is shown in Fig. S3C. In particular, 3 amino acid DEMs (2-phenylglycine, L-homocysteine, D-asparagine) that were upregulated in the OG root exudates had the highest VIP values (> 1) (Fig. S3D). Moreover, 2 of the significantly upregulated DEMs in the OG root exudates (L-leucyl-L-alanine and cordycepin) were found to be significantly positively correlated with the abundance of B68 (Table S6). In addition, as signaling molecules, cinnamic acid derivatives (such as the DEMs sodium ferulate and sinapinic acid) may play vital roles in the chemotaxis of rhizosphere bacteria. Therefore, these 4 amino acids (2-phenylglycine, L-homocysteine, D-asparagine and L-leucyl-L-alanine), 1 alkaloid (cordycepin) and 2 cinnamic acid derivatives (sodium ferulate and sinapinic acid) underwent in vitro assays. The four amino acids at 0.02–0.5mM, cordycepin at 0.01–0.5mM, sodium ferulate at 0.0–0.1mM, and sinapinic acid at a high concentration of 0.1mM significantly promoted B68 chemotaxis and biofilm formation (Fig. 2H, I).

### Effects of B68 inoculation on rhizosphere bacterial community

**Fig. 3:**
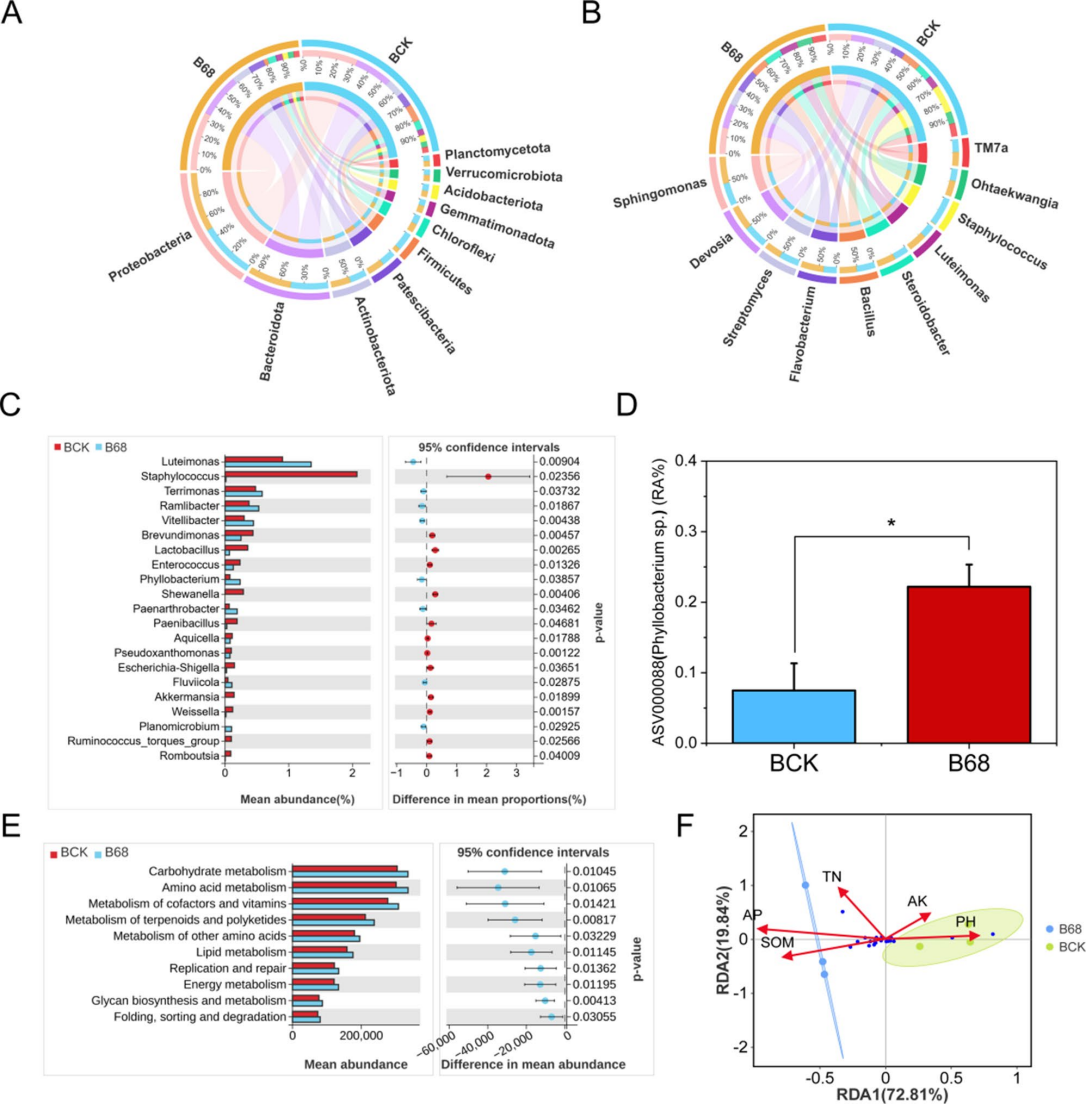
Effects of B68 inoculation on bacterial community composition and function. Relative abundances of dominant bacterial (**A**) phyla and (**B**) genera in *L. chinensis* rhizosphere soil. (**C**) Significant differences in mean abundances of genera between BCK and B68 groups (Welch’s t test). (**D**) Relative abundance of ASV000088 (*Phyllobacterium* sp. B68) after B68 inoculation. (**E**) Significant differences in mean abundances of genus functions between BCK and B68 groups. (**F**) Redundancy analysis of the abundances of ASVs and environmental factors. BCK, no inoculation; B68, inoculation with *Phyllobacterium* sp. B68

After B68 inoculation (compared to no inoculation; both groups had undergone mowing treatment), 741,508 ASVs in the 6 rhizosphere soil samples (6754 to 7629 per sample). The most abundant bacterial phyla were Proteobacteria (25.57%), Bacteroidota (19.60%), Patescibacteria (10.62%) and Actinobacteriota (9.32%) (Fig. 3A). The most abundant bacterial genera were Sphingomonas, Devosia, Streptomyces, Flavobacterium and Bacillus (Fig. 3B). B68 inoculation (compared to non-inoculation) significantly enriched Terrimonas, Ramlibacter, Phyllobacterium (ASV000088) and Luteimonas (Fig. 3C). In addition, ASV000088 had 100% nucleotide similarity match with the sequences of *Phyllobacterium* sp. B68. After inoculation, the abundance of ASV000088 was significantly higher than that non-inoculation (Fig. 3D), which indicates that *Phyllobacterium* sp. B68 can largely achieve successful colonization in the rhizosphere of *L. chinensis*. Based on the PICRUSt2 functional predictions, B68 inoculation enriched almost all metabolic pathways, including Amino acid metabolism, Energy metabolism and Carbohydrate metabolism (Fig. 3E). A redundancy analysis (RDA) revealed a significant correlation between the rhizosphere bacteria and rhizosphere soil characteristics after B68 inoculation. Additionally, the total N (*p* = 0.0278) and available P (*p* = 0.0389) were positively correlated with the bacterial community (Fig. 3F).

### B68 inoculation promoted the growth of *L. chinensis*

**Fig. 4:**
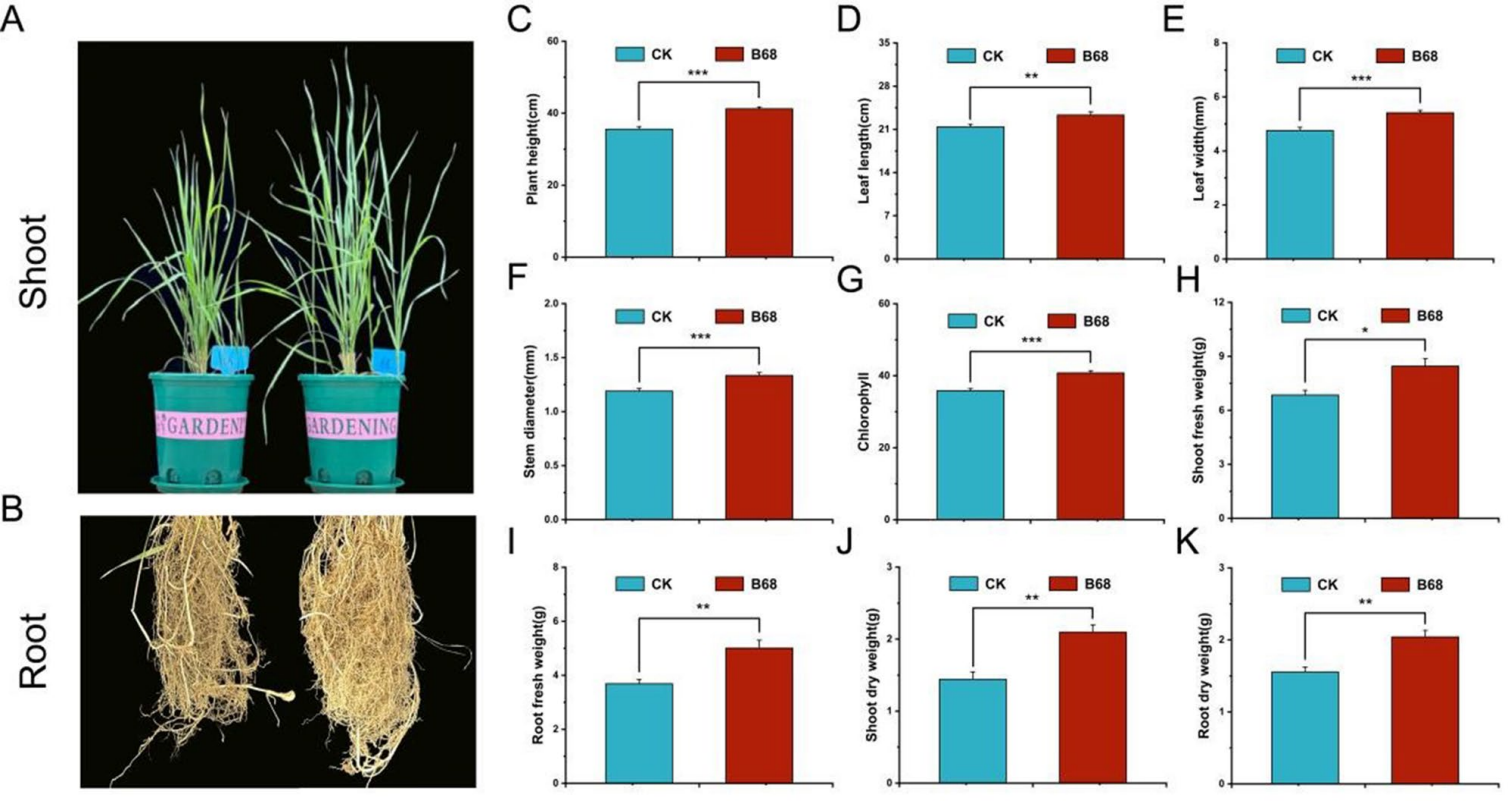
B68 inoculation promoted *L. chinensis* growth. (**A, B**) Effects of B68 inoculation on *L. chinensis* morphology. (**C–K**) Plant height, leaf length, leaf width, stem diameter, chlorophyll, fresh shoot weight, fresh root weight, dry shoot weight and dry root weight. BCK, non-inoculation; B68, inoculation with *Phyllobacterium* sp. B68. Bar chart shows mean ± SE. *p < 0.05; **p < 0.01; ***p < 0.001

At 4 weeks after B68 inoculation (compared to no inoculation), *L. chinensis* height, stem diameter, leaf length, chlorophyll content, leaf width, fresh leaf weight, fresh root weight, dry shoot weight, and dry root weight were significantly increased by 15.96%, 12.21%, 9.00%, 13.83%, 13.89%, 23.45%, 35.92%, 45.49%, and 31.57%, respectively (Fig. 4).

### Effects of B68 inoculation on phytohormones, nutrients and enzymatic activity

**Fig. 5:**
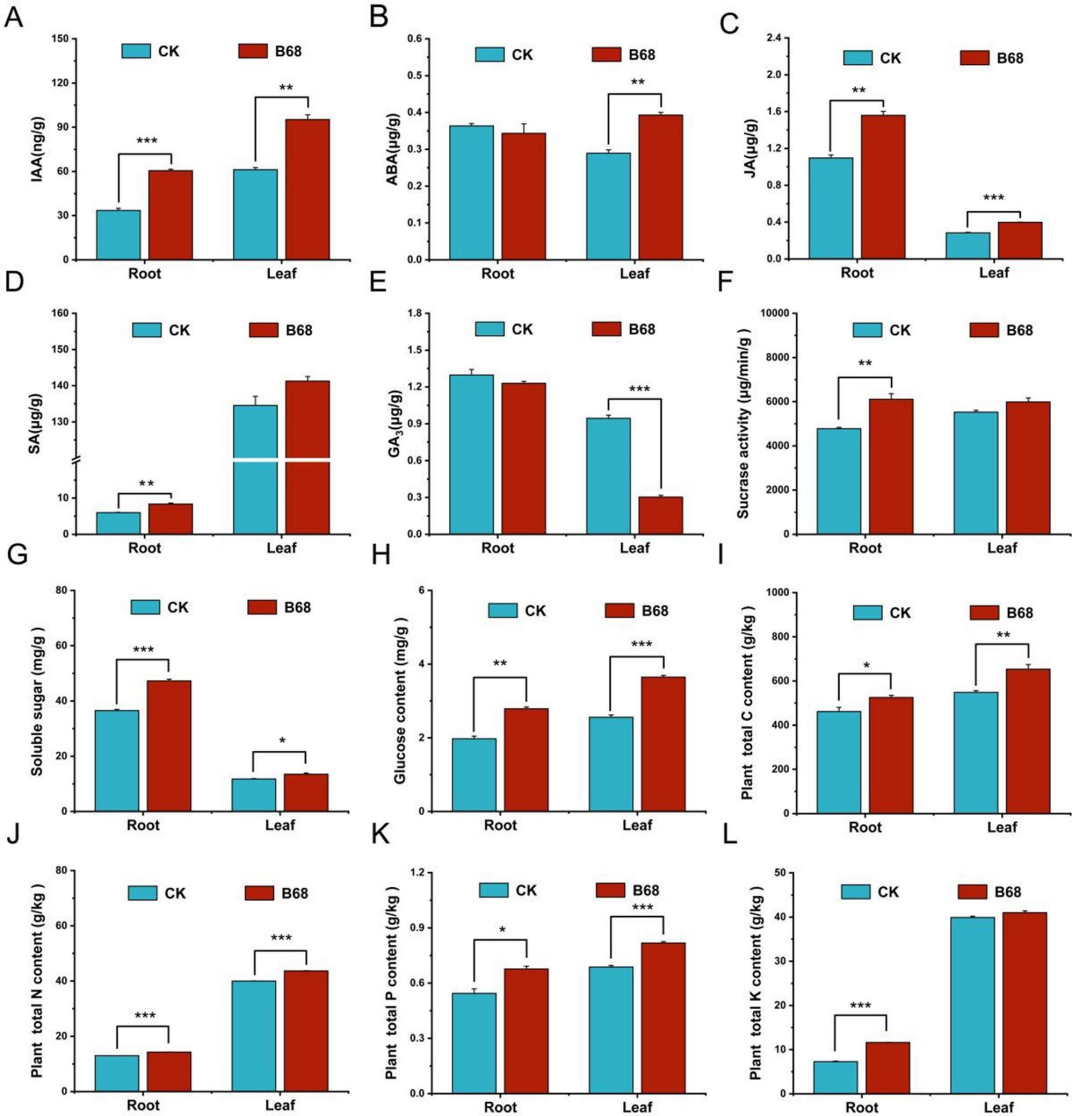
B68 inoculation affects phytohormone and nutrient content in L. chinensis leaves and roots. (**A–L**) Auxin (IAA), abscisic acid (ABA), jasmonic acid (JA), salicylic acid (SA), gibberellin (GA3), sucrase activity, soluble sugar, glucose, total carbon, total nitrogen, total phosphorus, total potassium content in roots and leaves. CK, non-inoculation; B68, inoculation with *Phyllobacterium* sp. B68. Bar chart shows mean ± SE. *p < 0.05; **p < 0.01; ***p < 0.001

B68 inoculation significantly increased IAA (55.70–80.18%) and JA (39.94–42.24%) in leaves and roots (Fig. 5A, C); soluble sugar (14.48–29.56%), glucose (41.14–42.50%), plant total C (13.92–19.28%), total N (9.16–10.22%), and total P (18.96–24.34%) in leaves and roots (Fig. 5G–K); ABA in leaves (Fig. 5B), and SA, sucrase activity, and plant total K in roots (Fig. 5D, F, L). B68 inoculation also significantly activated the antioxidant enzyme system, involving increased SOD (57.29%), CAT (95.82%), and POD (6.53%) (Fig. S4B–D). B68 inoculation also significantly increased RuBisCO activity in leaves (185.86%) (Fig. S4E), and PPO, PAL and LOX in leaves (Fig. S4F–H). All of the growth promotion results suggest that B68 inoculation increased antioxidant enzyme activity, plant growth, and N/P nutrient acquisition.

### Characterization of plant transcriptome in response to B68 inoculation

#### Transcriptomic sequencing and DEG identification

A total of 506,275,828 clean reads were obtained. The percentages of GC and Q30 were 52.91-54.86% and 88.93–90.57%, respectively (Table S7). Between the inoculated samples and on inoculated controls, 14,501 (8344 up-regulated and 6157 down regulated) in the leaves (Fig. S5A, C) and 17,971 genes were differentially expressed (10330 up-regulated and 7641 down regulated) in the roots (Fig. S5B, C). Differentially expressed genes (DEGs) threshold: Padj < 0.05;|log2FC| > 1 (Fig. S5B).

GO and KEGG analyses were used to explore the biological functions of DEGs between the B68-inoculated and non-inoculated groups. Regarding GO terms in leaves, the most significantly enriched were phosphorylation, cellular protein modification process and protein modification process (Fig. S5D). Regarding GO terms in roots, the most DEGs participated in carbohydrate metabolic process, structural constituent of cell wall and oxidoreduction-driven active transmembrane transporter activity (Fig. S5E). The dominant KEGG pathways in root were Energy metabolism and Lipid metabolism (Fig. S5F) and the most DEGs were significantly and specifically enriched in Plant hormone signal transduction, Photosynthesis – antenna proteins, and Starch and sucrose metabolism in leaves (Fig. S5G).

### B68 inoculation affected plant hormone signal transduction

**Fig. 6:**
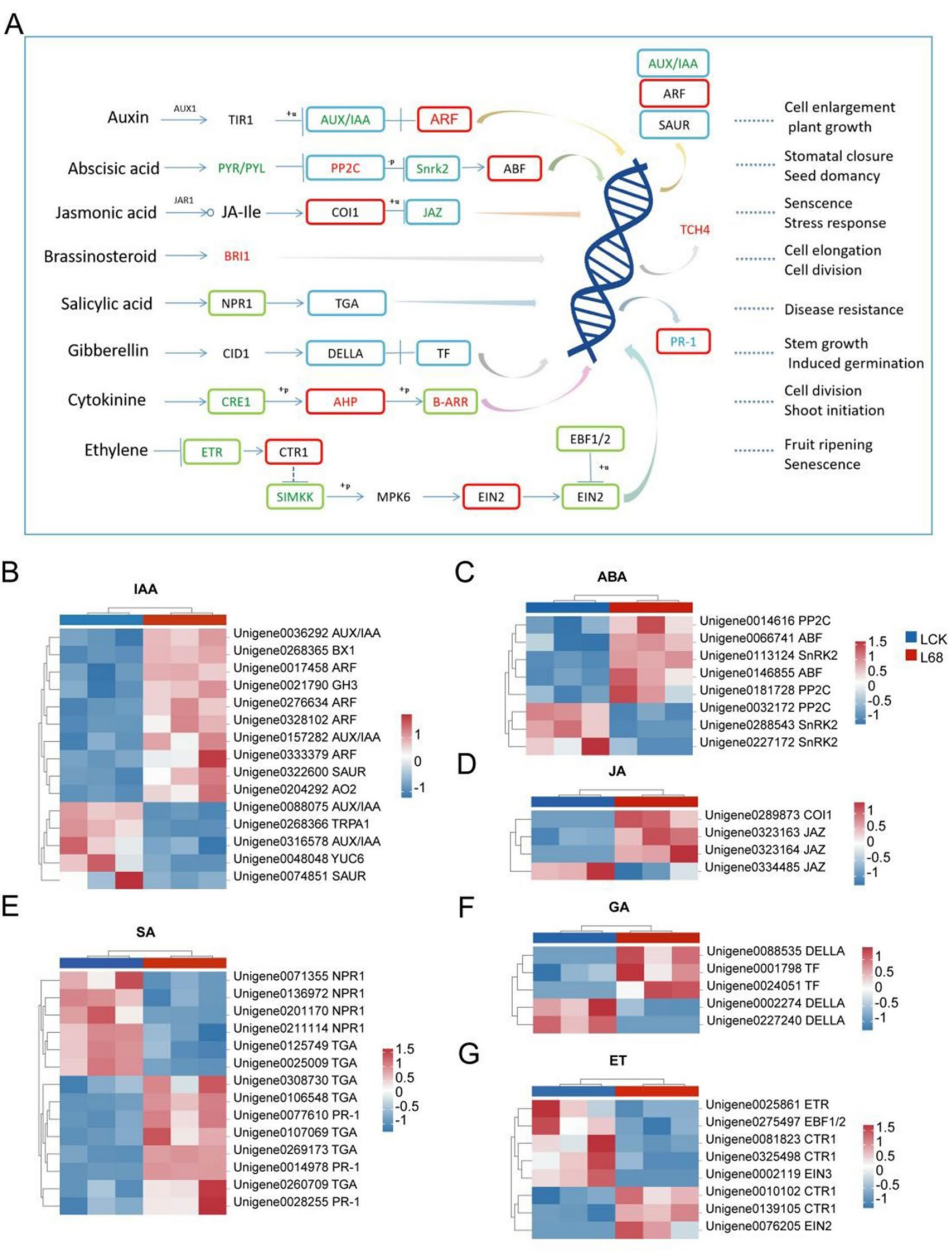
Expression of DEGs involved in phytohormone signaling. (**A**) Schematic diagram of phytohormone signaling pathways. Red, green, and blue text indicate upregulated, downregulated, and both upregulated and downregulated DEGs, respectively, in roots. Red, green, and blue borders indicate upregulated, downregulated, and both upregulated and downregulated DEGs, respectively, in leaves. (**B–G**) Expression of DEGs related to auxin (IAA), abscisic acid (ABA), jasmonic acid (JA), salicylic acid (SA), gibberellin (GA3), and ethylene (ET) pathways in leaves. LCK, non-inoculated leaves; L68, B68-inoculated leaves

To investigate the effects of B68 inoculation on phytohormone signaling in *L. chinensis* leaves and roots, DEG expression related to IAA, SA, JA, ABA, cytokinin, ethylene, gibberellin, and brassinosteroid pathways was analyzed (Fig. 6, Fig. S6). In the IAA pathway, 15 and 8 auxin synthesis and signal transduction related DEGs in leaves and roots, respectively, were differentially expressed. These included auxin synthesis and response related genes, Auxin/indoleacetic acid (AUX/IAA) genes, and Gretchen Hagen 3 (GH3) genes, among which, Auxin response factor (ARF) genes and several Small auxin-up RNA (SAUR) genes in both leaves and roots were upregulated (Fig. 6B, S6).

In the SA pathway, 5 TGACG-binding factor (TGA) genes and 3 Pathogenesis-related (PR-1) genes in leaves were upregulated, while 2 TGA genes and 4 Nonexpressor of pathogenesis-related gene 1 (NPR1) in leaves were downregulated (Fig. 6E). 14 PR-related DEGs in roots were differentially expressed (Fig. S6B). In the JA pathway, the 3 Jasmonate ZIM-domain (JAZ) genes and 1 Coronatine Insensitive1 (COI1) gene in leaves were differentially expressed. All JAZ genes in roots were downregulated (Fig. 6D, S6E). In the ABA pathway, 8 and 3 ABA signaling-related DEGs in roots and leaves, respectively, were differentially expressed. Among these, 2 ABRE binding factor (ABF) genes in leaves were upregulated, and 2 Protein phosphatase 2 C (PP2C) genes in leaves and roots were upregulated (Fig. 6C, S6F). In the cytokinin pathway, all the *Arabidopsis* histidine phosphotransfer protein (AHP) genes in both leaves and roots were upregulated (Fig. 6A, S6D). In the ethylene pathway, 2 CTR1 genes in leaves were upregulated (Fig. 6G). In the gibberellin pathway, 3 DELLA proteins and 2 transcription factors in leaves were differentially expressed (Fig. 6F). In the brassinosteroid pathway, 3 DEGs in roots were upregulated (Fig. S6G). Thus, B68 inoculation may promote plant growth by regulating the interactions between phytohormones.

### B68 inoculation altered the expression of DEGs related to sugar transport and metabolism

Through the enrichment analysis of gene function, it was found that strain B68 induced the changes of DEGs encoding starch and sucrose metabolism in leaves and roots. In addition, there were 5 DEGs related to sugar transport in leaves were upregulated (Fig. S7A). 5 DEGs related to sugar transport were changed in root, among which 3 DEGs were up-regulated, and 2 were down-regulated (Fig. S7B). Thus, B68 inoculation may affect plant sugar transport and metabolism.

### B68 inoculation altered the expression of DEGs related to nutrient uptake and metabolism

Expressions of high-affinity transporters associated with potassium, nitrate, iron and inorganic phosphate were differentially expressed. The expression of 4 and 5 nitrate transporter related DEGs in roots and leaves, respectively, were upregulated by B68 inoculation (Fig. S7A, B), and 23 and 14 nitrogen metabolism related DEGs in roots and leaves, respectively, were differentially expressed (Fig. S7C, D). Many phosphate transporters were differentially expressed by B68 inoculation. 3 phosphate transporter in leaves were upregulated, whereas 6 in roots were downregulated (Fig. S7B). In addition, expressions of 20 and 11 potassium transporters and metabolism related DEGs were upregulated by B68 inoculation in roots and leaves, respectively (Fig. S7E, F). The expression of 5 iron transporter and metabolism related DEGs in roots were upregulated, and 6 were downregulated (Fig. S7H). However, all iron transporter and metabolism related DEGs in leaves were upregulated (Fig. S7G).

### B68 inoculation altered the expression of DEGs related to growth and development

**Fig. 7:**
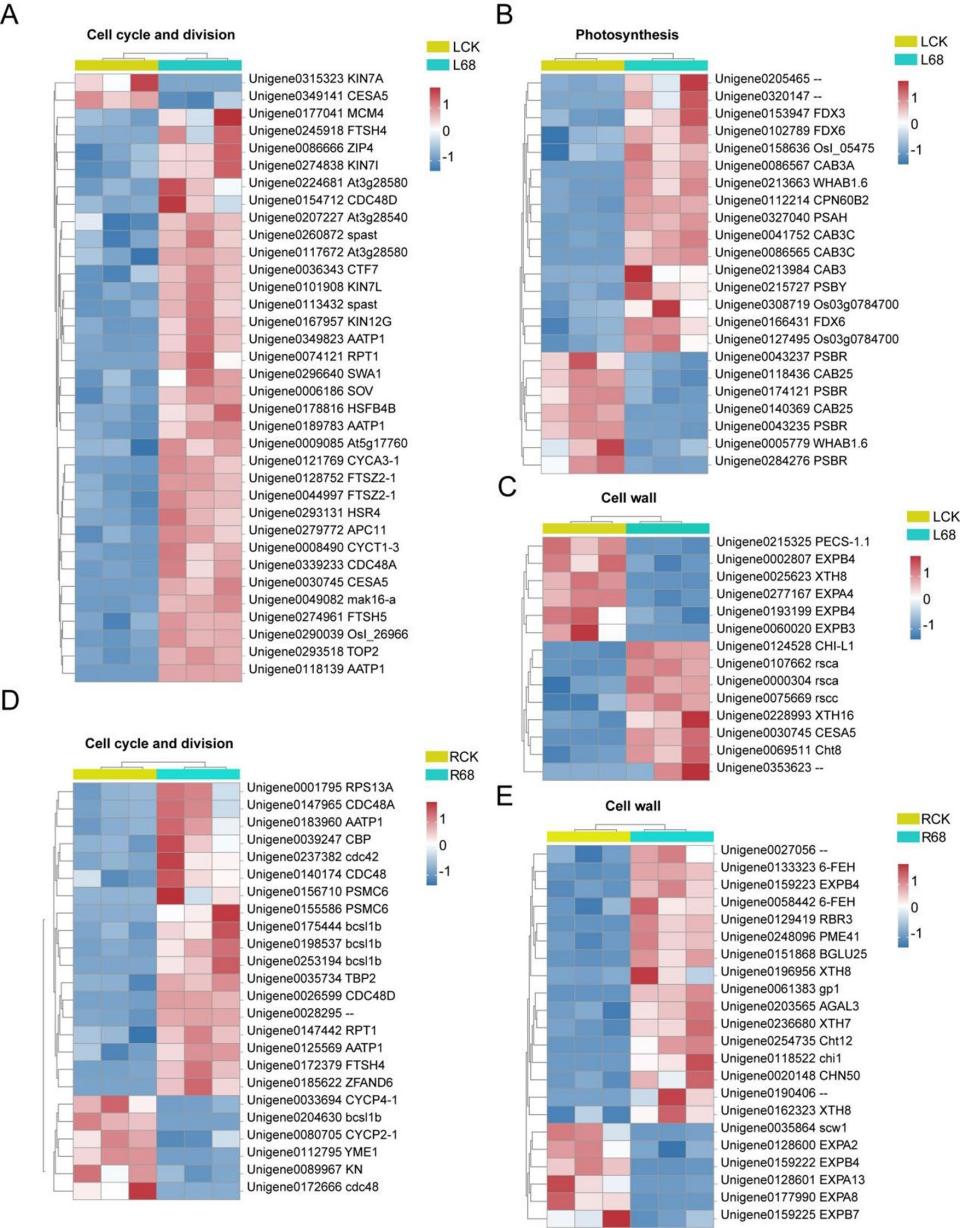
Expression of DEGs involved in plant growth and development. DEGs related to (**A, D**) cell cycle and cell division in roots and leaves, (**B**) photosynthetic electron transport chain and photosynthesis in leaves, and (**C, E**) cell wall synthesis, extension, and modification in roots and leaves. LCK, non-inoculated leaves; L68, B68-inoculated leaves; RCK, non-inoculated roots; R68, B68-inoculated roots

First, B68 inoculation affected the expression of photosynthesis-related genes (Fig. 7B). The most enriched were photosynthetic electron transport chain, light-harvesting, and photosynthesis related DEGs, including 6 photosystem II reaction center subunits, 2 photosystem I reaction center subunits, 7 photosynthethic electron transporter and 10 LHC genes. Among them, 7 photosynthethic electron transporter were significantly upregulated after B68 inoculation. In particular, the RuBisCO large subunit-binding protein (CPN60B2) gene was upregulated, which increases CO_2_-fixation efficiency. Second, B68 inoculation upregulated cell cycle and cell wall related genes in roots and leaves. The expression of 18 and 33 DEGs in roots and leaves related to cell cycle and cell division were upregulated by B68 inoculation (Fig. 7A, D). Regarding cell wall synthesis, extension, and modification, 16 and 8 DEGs in roots and leaves, respectively, were upregulated (Fig. 7C, E). Moreover, B68 inoculation also significantly upregulated several unigenes annotated as endotransglycosylase/hydrolases (XTHs) in leaves and roots (Fig. 7C, E).

### Characterization of plant root metabolome in response to B68 inoculation

After inoculation with B68, 76 DEMs were classified into 10 categories (Fig. S8A, S8B). B68 inoculation significantly enriched terpenoids, especially geranylgeraniol (9.17-fold compared to non-inoculated roots), betulinic acid (2.54-fold), and isomangiferolic acid (2.75-fold), and the phenolic acid O-feruloyl coumarin (2.43-fold). The 20 most enriched KEGG pathways included Aminoacyl-tRNA biosynthesis, ABC transporters, Glycine, serine and threonine metabolism, and Biosynthesis of secondary metabolites (Fig. S8C). This indicated that membrane transport and amino acid metabolism in *L. chinensis* roots play a crucial role in regulating plant growth and development.

### Integrated metabolomic and transcriptomic analysis

**Fig. 8:**
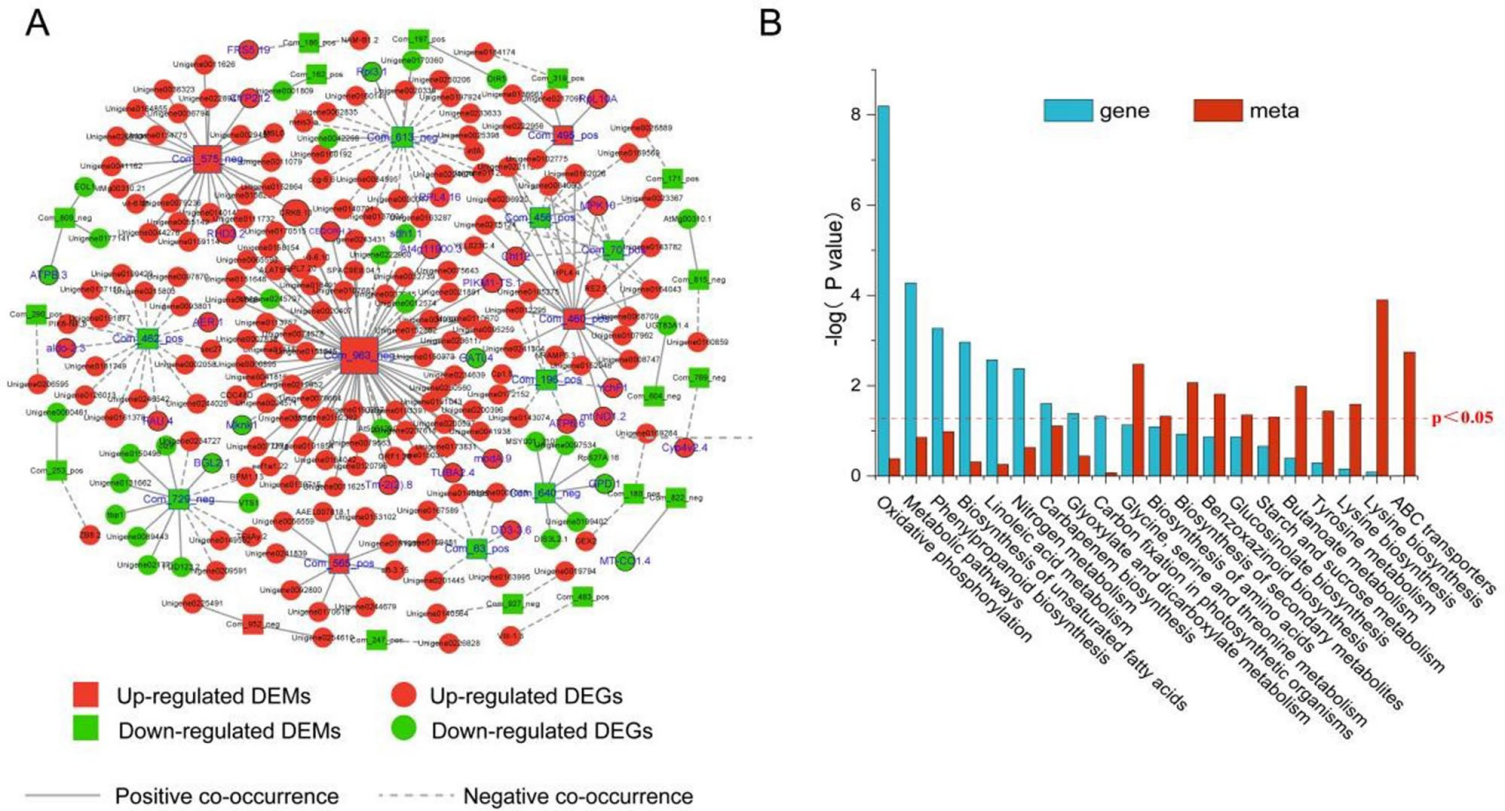
Integrated metabolome and transcriptome analysis. (**A**) Co-expression network analysis of the top 250 DEGs and DEMs (correlation coefficient > 0.95) in non-inoculated roots (RCK) vs. R68-inoculated roots. Network shows numerous connections between terpenoid DEMs and resistance-related DEGs. Gray solid and dotted lines are positive and negative interactions, respectively, between DEGs and DEMs. Red nodes indicate upregulated DEGs and DEMs, and green nodes indicate downregulated DEGs and DEMs. (**B**) KEGG analysis of DEGs and DEMs in non-inoculated roots (RCK) vs. R68-inoculated roots

The top 250 DEMs and DEGs (between the B68-inoculated and non-inoculated groups) were used in a co-expression network analysis (Fig. 8A). Several significantly enriched metabolites (terpenoid metabolites Com_963_neg and Com_575_neg and phenolic acid metabolite Com_460_pos) showed the strongest association with various DEGs (Fig. 8A). Among them, the CRK6 DEG was associated with Com_963_neg and Com_575_neg, so it may play a crucial role in regulating terpenoid metabolic signaling. In addition, KEGG analysis of the DEMs (*p* < 0.05) and DEGs (p_adj_<0.05) showed that they were significantly enriched mainly in oxidative phosphorylation, N metabolism, starch and sucrose metabolism, and phenylpropanoid biosynthesis (Fig. 8B). Thus, the analysis indicated that B68-induced changes in gene-regulated metabolites and metabolic pathways involved amino acid metabolism, energy metabolism and carbohydrate metabolism, thereby affecting *L. chinensis* growth and development.

#### RT-qPCR validation

RT-qPCR was used to verify the expression of DEGs in *L. chinensis* roots (Fig. S9A) and leaves (Fig. S9B) after B68 inoculation. The RT-qPCR and transcriptome data were similar for all assessed genes. Thus, the transcriptome data are accurate and reliable.

## Discussion

**Fig. 9:**
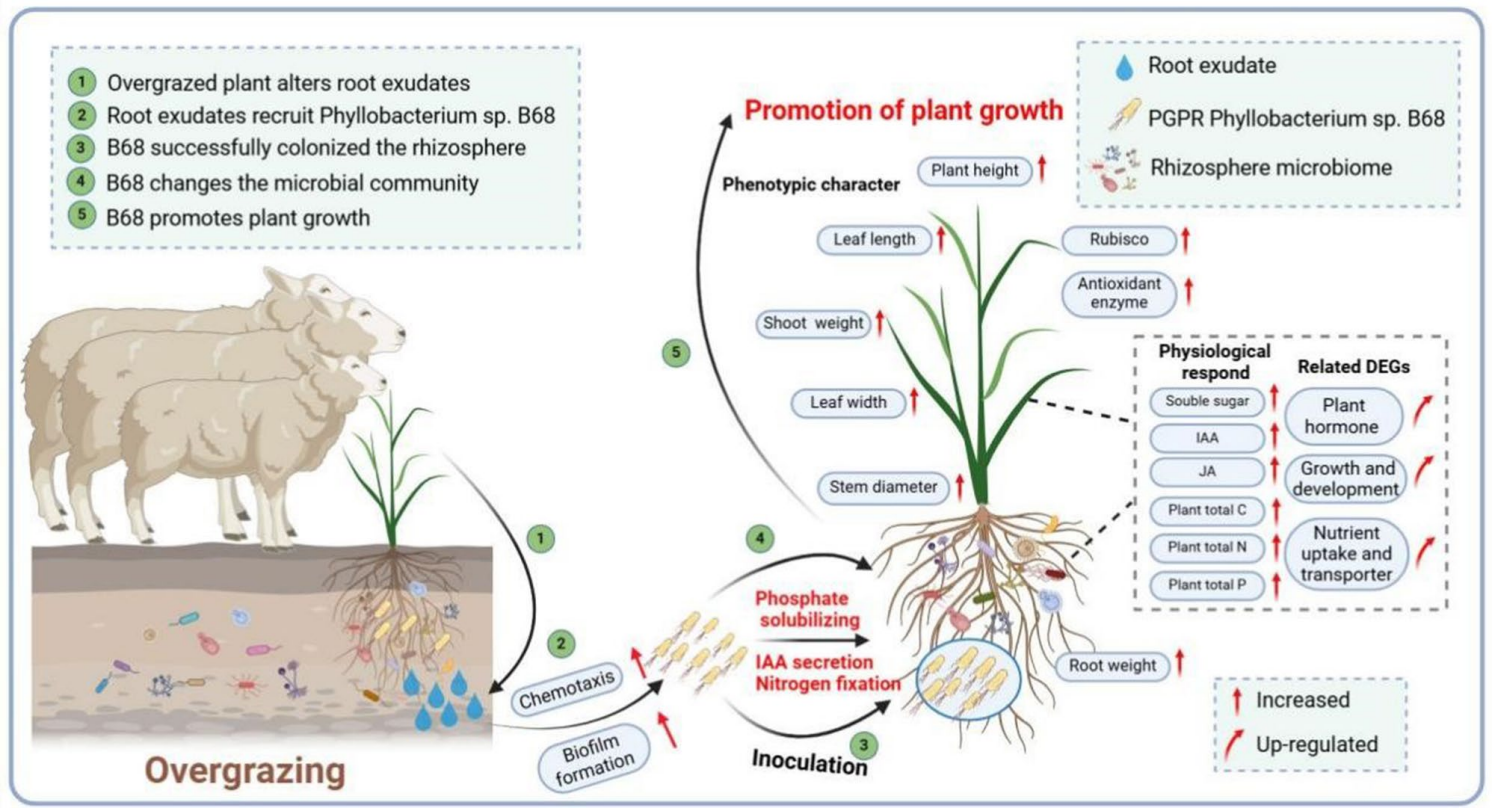
Mechanistic model of synergistic effects of grassland *L. chinensis* plant and beneficial rhizosphere bacteria under overgrazing (OG) stress. (1) Overgrazed L. chinensis alters root exudates to (2) recruit beneficial *Phyllobacterium* sp. B68 and (3) enhance B68 rhizosphere colonization by promoting B68 chemotaxis and biofilm formation. (4) Inoculation with B68 (which could solve phosphorus, fix nitrogen and produce IAA) reshaped the rhizosphere microbial community, leading to plant physiological and phenotypic changes, and (5) systemically upregulated genes related to multiple facets of growth and nutrient metabolism to promote plant growth and development

Plants have developed mechanisms to attract specific PGPR to their rhizosphere from the surrounding soil, including by regulating their root exudates [49]. PGPR influence host plant traits such as nutrition absorption and transportation, growth and development, and stress resistance [49]. Hence, it is essential to investigate these beneficial microbes and explore their growth-promoting mechanisms. In this study, grassland *L. chinensis* plants with long-term OG history in a typical steppe ecosystem improved their adaptation to OG stress by altering root exudates to recruit specific bacterial taxa, including PGPR *Phyllobacterium* sp. B68. Inoculated B68 may successfully colonized the rhizosphere, reshaped the rhizosphere bacterial community, altered plant phenotypes, and systemically regulated plant gene expression (related to nutrient metabolism and growth and development) to promote *L. chinensis* growth (Fig. 9).

### Effects of long-term overgrazing on rhizosphere microbial communities

In this study, we analyzed plant-microbe interactions in grassland plants L. chinensis with long-term overgrazing histories in a typical steppe ecosystem. The results showed that long-term OG significantly reshaped the *L. chinensis* rhizosphere microbial community and reduced the alpha diversity (Simpson index) (Fig. 2). The reduced diversity suggests that *L. chinensis* may selectively recruit specific rhizosphere microbes [6]. Grazing impacts soil microbial communities through various mechanisms [50, 51]. Livestock consume aboveground biomass and return nearly half of it as feces, which are rich in labile organic substrates and nutrients, potentially favoring rapid bacterial growth [52]. KEGG analysis showed that OG enriched starch and sucrose metabolism, ABC transporters, and quorum sensing (Fig. 2E). Carbohydrate metabolism and energy metabolism are fundamental metabolic pathways, creating precursors and energy sources for use in stress responses and secondary metabolism processes. ABC transporters are transmembrane proteins that transport metabolites such as amino acids, carbohydrates, and inorganic ions (Finkenwirth and Eitinger, 2019). Thus, OG may promote the synthesis and transport of carbohydrate metabolites, which is crucial for promoting plant growth and development and enhancing stress responses [53].

Grazing significantly changes nutrient cycling and utilization through trampling, defoliation, and manure deposition [54]. The effects of beneficial microbes on plant N and P uptake and use efficiency has been extensively studied [55, 56]. Our study showed that *L. chinensis* with OG history altered microbial community function and improved rhizosphere soil microbial N and P cycling (Fig. 2F and G; Fig. S1C, S1D). Similarly, Liu et al., (2023) [55] demonstrated that grazing promotes soil microbial P cycling and phosphate metabolism. This suggest that OG facilitates insoluble phosphate dissolution in the rhizosphere soil, thus providing P sources for plants, and affecting N fixation, ammonification, nitrification, and denitrification [57]. This constitutes a key mechanism by which grazing promotes N and P cycling in rhizosphere soil.

### *L. chinensis* under OG stress recruit beneficial rhizosphere bacteria by altering root exudates

Numerous studies have demonstrated that plants can selectively recruit beneficial rhizosphere soil microbes by altering root exudates, such as amino acids and secondary metabolites, in response to biotic and abiotic stresses [58]. These exudates or photosynthetic products serve as nutritional sources and chemical attractors for rhizosphere microbes, facilitating bacterial colonization and symbioses with plant roots through chemotaxis and biofilm formation [59, 60]. For instance, organic acids in cucumber root exudates can recruit Comamonadaceae [61]. Similarly, *Arabidopsis thaliana* secretes organic acids and amino acids to recruit specific *Pseudomonas* sp. to cope with foliar pathogen Pst [62].

The PGPR *Phyllobacterium* sp. B68 was only found in the OG rhizosphere soil samples (Fig. 2D). Additionally, cordycepin and L-leucyl-L-alanine were significantly upregulated DEMs in upregulated OG root exudates and were positively correlated with the abundance of B68 (Table S6). These findings suggest that specific bacteria such as B68 can be recruited by altering specific root exudates in order to enhance *L. chinensis* adaptability under OG stress. Bacteria can respond to chemotactic signals in root exudates (chemotaxis), primarily affecting colonization [63]. Studies have shown that *Panax notoginseng* increases cinnamic acid secretion to promote the chemotaxis of beneficial *Burkholderia* B36, which increases growth to alleviate autotoxic ginsenoside stress [64]. Similarly, in our study, 4 amino acids, 1 alkaloid, and 2 cinnamic acid derivatives that accumulated in *L. chinensis* root exudates under OG stress (Fig. S3D) significantly stimulated B68 chemotaxis and biofilm formation (within root exudate concentration ranges) (Fig. 2H, I). Thus, OG may trigger a “cry for help” strategy that allows plants to actively seek assistance from beneficial microbes to cope with stress.

### PGPR inoculation reshapes plant rhizosphere microbial communities

B68 inoculation significantly enriched 10 genera, most of which are beneficial to plants (Fig. 3C). For example, Ramlibacter has P-solubilizing activity and promotes plant growth [65]. Luteimonas degrades complex organic compounds, thereby enhancing N and P uptake and promoting early plant development [66]. Phyllobacterium has reasonable plant growth-promoting abilities [67, 68].

In general, high-abundance soil microbes have important ecological functions, while low-abundance microbes exhibit lower metabolic activity but can help to accommodate more microbes with limited nutrients, which participate in soil nutrient conversion, bioremediation, and improve plant growth and stress tolerance [69, 70]. Therefore, low-abundance Phyllobacterium detected only in OG conditions, was selected for subsequent pot experiments. Notably, *Phyllobacterium* sp. B68 inoculation (compared to non-inoculation) significantly increased abundance of Phyllobacterium in rhizosphere soil and had 100% sequence similarity with ASV000088 (*Phyllobacterium* sp. B68), which indicates that *Phyllobacterium* sp. B68 may successfully colonize the rhizosphere (Fig. 3C, D), indicating its beneficial effect on plants.

According to PICRUSt analysis of *L. chinensis* rhizosphere bacteria, B68 inoculation enriched several metabolic pathways (Fig. 3E). These results align with previous findings that phosphate-solubilizing bacteria inoculation can enrich metabolic pathways related to bacterial migration, amino acid metabolism, and C metabolism [71]. Metabolites associated with these pathways are secreted and can serve as N and C sources for soil microbes [72]. Enhanced C metabolism can increase intracellular energy production, supporting *L. chinensis* growth. These data further confirm that B68 inoculation may influence plant growth by modifying microbial metabolic activities. Therefore, incorporating beneficial flora in plants could be a strategy to improve plant growth.

### PGPR inoculation promotes plant growth and development

PGPR promote plant growth through P solubilization, N fixation, phytohormone production, and systemic resistance induction [73]. PGPR can also increase photosynthetic efficiency, and chlorophyll content [74]. Although PGPR inoculation promotes growth in Poaceae family plants, such as maize [75], wheat [76], rice, and sugarcane [77], there are few studies on the growth-promoting effects and regulatory mechanisms of PGPR on *L. chinensis*. Our study showed that PGPR B68 inoculation significantly increased plant growth (Fig. 4), which was similar to the results in previous studies [78, 79]. This increase may be attributable to IAA secretion, N fixation, and P solubilization by PGPR. PGPR-induced root development, increased shoot biomass, and plant growth promotion have been shown to be related to IAA synthesis [80, 81]. Bacteria with N fixation and P solubilization capacities significantly increased seed germination, root and shoot length, biomass, and plant growth [82, 83]. Thus, the enhanced *L. chinensis* growth observed upon B68 inoculation may be due to B68’s IAA secretion, N fixation, and P solubilization capacities (Fig. S2; Table S3).

In our study, we observed that B68 inoculation systemically regulated the transcription of key genes involved in plant growth and development (Fig. 7). In particular, B68 inoculation upregulated many cyclins and checkpoint genes regulating the cell cycle in roots and leaves (Fig. 7A, D), promoting mitosis by regulating cell cycle and protein synthesis, thereby improving plant growth. B68 inoculation also significantly upregulated several unigenes annotated as endotransglycosylase/hydrolases (XTHs; which have been implicated in cell wall loosening [84] and other genes related to cell wall modification in roots and leaves (Fig. 7C, E), suggesting that B68 increases cell wall flexibility. These cell wall modification-related DEGs also participate in various plant–microbe interactions [85]. Thus, B68 inoculation facilitates cell wall loosening, expansion, and elongation, leading to cell division and enhancing cell plasticity, promoting plant growth.

It is well known that increased chlorophyll content enhances light energy absorption and utilization, thereby boosting photosynthesis [86]. Beneficial microbial inoculants increase plant growth, chlorophyll content, and photosynthetic capacity [87]. Soluble sugars are energy storage substances and can play crucial roles as signal substances in plant–microbe interactions under stress [88]. B68 inoculation increased chlorophyll content (Fig. 4G), RuBisCO activity (Fig. S4E) and upregulated the RuBisCO large subunit-binding protein (CPN60B2) (Fig. 7B), indicating improved photosynthetic product accumulation in leaves. Our study also found that B68 inoculation increased *L. chinensis* photosynthetic performance by regulating photosynthetic electron transport, as the PetH gene was upregulated 2–53-fold in leaves; the PetH gene also helps to maintain the cellular balance of oxidized and reduced glutathione [89]. Additionally, this B68-induced increase in soluble sugar (Fig. 5G) and glucose (Fig. 5H) may be partly attributable to a higher net photosynthesis rate [90], further affecting N assimilation [91]. In plants, sugar transport is controlled by transport tissues and a variety of sugar transporters. B68 inoculation significantly upregulated 6 and 2 genes involved in sugar transport and metabolism in leaves and roots, respectively (Fig. S7A, B), suggesting that B68 enhanced sugar accumulation in *L. chinensis* by facilitating sugar transport. Thus, B68 improves photosynthetic capacity, promotes growth, and helps plants adapt to the environment by accumulating sugars in leaves and roots.

PGPR inoculation significantly increases antioxidant enzyme activities (SOD, CAT, and POD) [92]. Increased antioxidant enzyme activities can indicate PGPR-induced plant resistance, as they stabilize membrane permeability, reduce membrane peroxidation damage, and improve photosynthesis [93]. Similarly, in our study, SOD, CAT, and POD activities significantly increased by 57.29%, 95.82% and 6.53%, respectively, in *L. chinensis* leaves after B68 inoculation (Fig. S4B–D), indicating increases in antioxidant enzyme activities, regeneration ability, and resistance to mowing treatment.

### PGPR inoculation regulates plant nutrient uptake, nutrient transport, and hormone signaling

There are extensive studies showing that PGPR enhance plant nutrient use efficiency [94, 95]. Our results showed that B68 inoculation significantly improved *L. chinensis* growth and C/N/P acquisition (Fig. 5I–L), which is similar to previous findings [96]. Plants can alter their nutrient acquisition to cope with environmental changes, which is controlled by N and P signaling pathways [97]. These signaling pathways also regulate symbiosis involving beneficial microbes [98], thereby promoting plant growth and development [99].

Numerous interactions between plant-associated microbes and plants affect host access to N, P, and K [100]. Our study showed that B68 inoculation upregulated several genes related to phosphate transport and metabolism in leaves and roots (Fig. 5A, B). This suggests that B68 not only aids in releasing insoluble P but also activates systemic P signaling in leaves and roots, thereby enhancing P absorption and promoting its recycling between different tissues [21]. Additionally, B68 inoculation strongly upregulated 5 and 3 nitrate transporter genes in leaves and roots, respectively (Fig. S7A, B). These genes may play a crucial role in responding to systemic signals circulating between roots and shoots, and promote plant growth. These genes may be part of the PGPR-mediated mechanism regulating N signaling for root development [101]. Furthermore, B68 inoculation regulated genes related to N metabolism in leaves and roots (Fig. S7C, D), indicating that B68 promotes N metabolism in plants. *Phyllobacterium brassicacearum* STM196 inoculation dramatically upregulates nitrate transporter 2 (NRT2) family genes in plants [102], supporting our findings that B68 inoculation upregulated most of the DEGs involved in N acquisition, transport, and metabolism (Fig. S7), which further demonstrates that B68 enhances N uptake by roots and modifies N acquisition and metabolism pathways in leaves.

PGPR actively regulate multiple physiological processes, participating in plant growth by controlling endogenous phytohormones, nutrient balance, and plant signal molecule secretion [103]. PGPR promote plant growth by regulating homeostasis of phytohormones such as auxin, ABA, SA, and gibberellin [104, 105]. PGPR inoculation mainly affects root development by synthesizing auxin in root tissues, which increases root surface area, shoot biomass, and nutrient absorption capacity [106]. Similarly, B68 inoculation significantly increased IAA (55.70–80.18%) and JA (39.94–42.24%) in leaves and roots (Fig. 4A, C), ABA in leaves (35.70%), and SA in roots (39.17%) (Fig. 5B, D).

Phytohormone signaling and crosstalk play crucial regulatory roles in plant development, flowering, fruiting, and tissue differentiation [107]. Auxin-producing PGPR regulate auxin distribution and transport in plant tissue [108], so PGPR inoculation may increase endogenous auxin in plants, promoting plant growth. The synergistic action of various phytohormones improves plant growth and development [109]. B68 inoculation significantly upregulated several key genes in the IAA, ABA, JA, SA, cytokinin, and ethylene pathways (Fig. 6; Fig. S6). B68 inoculation likely exerted pro-growth effects through these hormone signaling pathways, with IAA playing a dominant role. In the auxin signaling pathway, AUX, ARF, and several IAA genes in roots and leaves were upregulated (Fig. 6; Fig. S6). This is further supported by research showing that PGPR inoculation regulates gene expression related to multiple aspects of growth and development, nutrient uptake and transport, and phytohormone signaling, particularly the auxin response pathway [22].

Changes in the rhizosphere microbial have been shown to affect the expression of DEGs related to nutrient absorption. Beneficial microbes regulate plant gene expression by producing auxin, secreting secondary metabolites, and altering soil nutrients to influence plant development [110]. Our study is consistent with reports showing that PGPR inoculation regulates endogenous phytohormone homeostasis and promotes plant growth while also regulating nutrient balance and increasing N fixation and P acquisition [111, 112]. Collectively, PGPR B68 promote *L. chinensis* growth by enhancing nutrient uptake and transport, maintaining hormone homeostasis, and altering the expression of genes related to plant growth and nutrient metabolism.

## Conclusions

This study illustrates how *L. chinensis* adapts to OG stress by recruiting specific PGPR. *L. chinensis* with OG history recruited the beneficial *Phyllobacterium* sp. B68 by regulating specific root exudates (such as amino acid and alkaloid compounds). Additionally, B68 inoculation leads to physiological and phenotypic changes in plants and systemically upregulated DEGs involved in multiple facets of growth and nutrient metabolism (such as nitrate, inorganic phosphate, and sugar transporters, N metabolism, cell division, cell wall modification, and photosynthesis) to promote plant growth. These findings showed that plants under OG stress increase the recruitment of beneficial bacteria to the rhizosphere and PGPR inoculation comprehensively regulates gene expression and metabolic pathways associated with plant growth and development, forming an integrated adaptive mechanism that helps plants more effectively cope with stress and improves plant adaptability and growth. Collectively, these findings deepen our understanding of plant–microbe synergies in grazing grassland ecosystems, optimizing grassland resource utilization, increasing yield, enhancing stress resistance, and promoting the sustainability of grassland ecosystems.

## Electronic supplementary material

Below is the link to the electronic supplementary material.

Supplementary Material 1

Supplementary Material 2

## Data Availability

Data is provided within the manuscript and supplementary information files. Morever raw sequencing data have been submitted to the National Center for Biotechnology Information (NCBI) Sequence Read Archive (SRA) database under the accession number PRJNA1116349, PRJNA1116623 and PRJNA1116777.
