# Supplementary material for: Synergistic effect of grassland plants and beneficial rhizosphere bacteria helps plants cope with overgrazing stress
Source: BMC Plant Biol. 2025 May 10;25:614. doi: 10.1186/s12870-025-06638-z (PMC12065256; doi:10.1186/s12870-025-06638-z)
Supplement: Supplementary file 2 — Supplementary Material 2 [file 12870_2025_6638_MOESM2_ESM.docx]

**Supplementary file**

**Supplementary Methods**

**Collection of rhizosphere soils and root exudates**

*L. chinensis* rhizosphere soils were collected as described by Chen et al. (2020) [1]. In each plot, 15 *L. chinensis* plants were carefully uprooted with a shovel, and the roots were shaken to remove bulk soil. The remaining soil on the root surfaces (approximately 1–3 mm thick) was considered rhizosphere soil and collected into sterile self-sealing bags using a hairbrush [2]. These 15 rhizosphere soil samples from each plot were then combined into one composite sample. *L. chinensis* root exudates were collected using a modified method based on Ren et al. (2008) [3] and Luo et al. (2020) [4]. *L. chinensis* plants were collected from NG and OG subsites, ensuring that the roots remained as intact as possible. After cleaning the roots with sterile deionized water, 20 *L. chinensis* plants with similar growth trends were placed in beakers containing Hoagland’s complete nutrient solution, with roots submerged for 3 days for regeneration. The beakers were wrapped in tinfoil to protect them from light and kept in a growth chamber at 28°C with aeration for 6 h/day to repair any root damage. After regeneration, the roots were transferred to beakers containing 1000 mL sterile distilled water and incubated at 28–30°C for 12 h. The collected root exudates were then filtered, concentrated to dry matter under reduced pressure, and stored at −80°C for metabolomic profiling [5].

**High-performance liquid chromatography (HPLC)-MS/MS-based metabolomic profiling of roots and root exudates**

*L. chinensis* root samples (100 mg) from the B68-inoculated and non-inoculated groups were ground to a fine powder using a mixer mill (Retsch GmbH, Düsseldorf, Germany) in liquid nitrogen. Metabolites were extracted at 4°C with 80% aqueous methanol, centrifuged for 20 min to remove solid particles, filtered (0.22-μm pore size), and analyzed by Gene Denovo Biotechnology Co., Ltd. (Guangzhou, China). Briefly, 2-μl samples were injected into a XSelect HSS T3 column (2.1×150 mm, 2.5 μm) at a flow rate of 0.4 mL/min. MS analysis involved electrospray ionization and the following parameters: temperature at 550°C, curtain gas at 35 psi, collision gas set to medium, ion spray voltage at -4500 V and 5500 V, ion source gas 1 at 60 psi, and ion source gas 2 at 60 psi. Detection using multiple reaction monitoring (MRM) was based on an in-house database. HPLC-MS/MS data files were integrated and peak-corrected using SCIEX OS v1.4 [6]. Additionally, root exudates (100 mL) from the NG and OG groups were lyophilized, dissolved in 80% methanol, and analyzed by HPLC-MS/MS using the same procedure.

**In vitro chemotaxis and biofilm formation assays**

To test the effects of 2-phenylglycine, l-homocysteine, d-asparagine, l-leucyl-l-alanine, cordycepin, sodium ferulate, and sinapinic acid on B68, five concentrations of each compound (0, 0.001, 0.01, 0.02, 0.05, 0.1, and 0.5 mM) were evaluated. First, the effects of the root exudate compounds on B68 chemotaxis were measured using modified capillary assays based on the protocol by Rudrappa et al. (2008) [7] and Gordillo et al. (2007) [8]. Briefly, a 25-gauge needle, attached to a 1-ml syringe containing 100 μl B68 suspension (10^8^ CFU/ml) or LB medium (negative control) was used as the chemotaxis capillary. After static incubation for 2 h at 30°C, the solution in the syringe was diluted, and the number of B68 cells was counted on LB agar. Second, the effects of the root exudate compounds on B68 biofilm formation were measured using biofilm formation assays [9,10]. B68 was grown until an optical density at 600 nm (OD600) of 1.0 was reached, centrifuged, washed, and resuspended in 1/2 MSgg (minimal salts, glutamate, and glycerol). Each well of a 48-well microtiter plate was inoculated with freshly prepared 1/2 MSgg medium and B68 suspension. Next, 10 μl root exudate compound or 1/2 MSgg medium (negative control) was added to each well. Each treatment had six replicates with four wells per replicate. After static incubation for 24 h at 30°C, adhered cells were stained with 0.1% crystal violet. Biofilm formation was quantified by measuring the OD_470_ of the solution in each well using a CFX96 ^TM^ Real-Time System (Bio-Rad).

**Phosphate solubilization assay**

The qualitative phosphate-solubilizing capacity of B68 was assessed by incubation on Pikovskaya (PKO) agar containing tricalcium phosphate at 30°C for 7 days. Formation of halo zones (clear zones) around the B68 colonies indicated phosphate-solubilizing activity. The halo zone size was measured and the phosphate solubilization index (PSI) was calculated as total diameter (colony + clear zone)/colony diameter [11]. The quantitative inorganic phosphate-solubilizing capacity of B68 was assessed based on the stannous chloride method with modifications [12]. This analysis used PKO medium in Erlenmeyer flasks containing 5 g/L phosphate inoculated with 1 mL B68 culture (OD_600_=1). The flasks were placed in a rotary shaker for 5 days (30°C, 180 rpm). Thereafter, the supernatant was obtained to measure the inorganic phosphate content based on the molybdenum blue method using a UV spectrophotometer. A standard curve was prepared using a standard series of diluted KH_2_PO_4_ (0.2, 0.4, 0.6, 0.8, and 1 mg P/L) [13].

**Indole-3-acetic acid (IAA) production assay**

The IAA production capacity of B68 was assessed as described by Chrastil (1976) [14]. Briefly, B68 was cultured in LB medium containing 100 mg/L L-tryptophan for 2 days on a rotary shaker (30°C,180 rpm). The culture supernatant (1mL) was mixed with 3mL Salkowski reagent (10.8 M H_2_SO_4_ and 4.5 g FeCl_3_ in 1000 mL distilled water) and incubated in the dark. After 30 min, the optical density (OD) was measured at 530 nm using a UV spectrophotometer. This experiment was replicated three times. The IAA stock solution (1000 µg/mL) was diluted to create a standard series (0, 10, 20, 30, 40, 50, 60, 70, 80, and 90 µg/mL) for calibration. IAA concentration was estimated using the standard curve, with the development of a pink color indicating IAA production (Glickmann and Dessaux 1995)[15].

**Nitrogenase activity assay**

Nitrogenase activity was detected using a double-antibody detection method involving an enzyme antibody (nitrogenase) and a horseradish peroxidase (HRP)-labeled antibody (Wang et al. 2024)[16]. This assay employs a double antibody sandwich technique to measure nitrogenase (NITS) levels in samples. Purified NITS antibodies were coated onto microporous plates to create solid-phase antibodies. Nitrogenase NITS antibodies were added to the plates, followed by HRP-labeled nitrogenase NITS antibodies, forming an antibody-antigen-enzyme-labeled antibody complex. After thorough washing, the substrate TMB was added for color development. TMB is catalyzed by HRP, turning blue and then yellow under acidic conditions. The color intensity is directly proportional to the NITS concentration in the sample. The concentration of NITS activity was calculated from a standard curve (OD_450_).

**Transcriptomic sequencing of *L. chinensis* leaves and roots**

Total RNA was extracted from *L. chinensis* leaves and roots in the B68-inoculated and non-inoculated groups using a TRIzol reagent kit (Invitrogen, Carlsbad, CA) following the manufacturer’s protocol. RNA integrity and concentration were assessed using a Qubit (Thermo Fisher Scientific, Waltham, MA) and Nanodrop (Thermo Fisher Scientific, Waltham, MA) accordingly. Samples with the highest RNA quality were selected for sequencing. RNAs with poly(A) tails were purified using oligonucleotide (dT) magnetic beads and fragmented into short sequences for cDNA synthesis. The library fragments were then purified using a QiaQuick PCR extraction kit (Qiagen, Venlo, The Netherlands). The ligation products were size-selected by agarose gel electrophoresis, PCR amplified, and sequenced using the Illumina Novaseq X Plus by Gene Denovo Biotechnology Co. (Guangzhou, China). Clean paired-end reads were obtained after processing, filtering, and quality control of the raw data. The filtered reads were aligned to the reference genome using TopHat, and the distribution of reads was analyzed.

**Metagenomic sequencing of *L. chinensis* rhizosphere soil**

Total DNA was extracted from the rhizosphere soil in the NG and OG groups using a Soil DNA Kit (Magen, Guangzhou, China). DNA concentration and quality were measured using Qubit and NanoDrop (Thermo Fisher Scientific, Waltham, MA). The genomic DNA was fragmented to construct a paired-end library. The library size distribution was analyzed using a 2100 Bioanalyzer (Agilent, Santa Clara, CA). Sequencing was performed using paired-end technology (PE150) on an Illumina NovaSeq 6000 sequencer by Gene Denovo Biotechnology Co., Ltd. (Guangzhou, China). Raw data were filtered using FASTP v0.18.0. Genes were predicted from the assembled contigs (>500 bp) using MetaGeneMark v3.38. Predicted genes ≥300 bp were pooled and clustered at ≥95% identity and 90% read coverage using CD-HIT v4.6 (Zhu et al. 2010; Fu et al. 2012)[17,18]. Predicted genes were aligned against the non-redundant genome databases of NCBI using DIAMOND v0.9.24 (Buchfink et al. 2015)[19]. The lowest common ancestor (LCA) algorithm in MEGAN v6.19.9 (Huson et al. 20)[20] was used to verify species annotation. Unigenes were annotated by alignment with various protein databases including NCBI Nr, KEGG, and eggNOG v4.5 using DIAMOND v0.9.24. Differential abundances of functional genes between NG and OG rhizosphere soil microbiomes were explored using Venn diagrams based on the R package VennDiagram (Chen et al. 2011)[21]. Alpha diversity indices, species, and functions were compared between groups using Welch’s t-tests in the R package Vegan.

**References**

Chen Y, Bonkowski M, Shen Y, Griffiths BS, Jiang Y, Wang X, Sun B. Root ethylene mediates rhizosphere microbial community reconstruction when chemically detecting cyanide produced by neighbouring plants. Microbiome. 2020; 8:1-17.

Philippot L, Raaijmakers JM, Lemanceau P, Van Der Putten WH. Going back to the roots: the microbial ecology of the rhizosphere. Nat Rev Microbiol. 2013; 11(11):789-799.

Ren L, Su S, Yang X, Xu Y, Huang Q, Shen Q. Intercropping with aerobic rice suppressed Fusarium wilt in watermelon. Soil Biol Biochem. 2008; 40(3):834-844.

Luo LF, Yang L, Yan ZX, Jiang BB, Li S, Huang HC, Liu YX, Zhu SS, Yang M. Ginsenosides in root exudates of Panax notoginseng drive the change of soil microbiota through carbon source different utilization. Plant Soil. 2020; 455:139-153.

Deng L, Luo L, Li Y, Wang L, Zhang J, Zi B, Ye C, Liu Y, Huang H, Mei X, Deng W. Autotoxic ginsenoside stress induces changes in root exudates to recruit the beneficial Burkholderia strain B36 as revealed by transcriptomic and metabolomic approaches. J Agric Food Chem. 2023; 71(11):4536-4549.

Want EJ, Masson P, Michopoulos F, Wilson ID, Theodoridis G, Plumb RS, Shockcor J, Loftus N, Holmes E, Nicholson JK. Global metabolic profiling of animal and human tissues via UPLC-MS. Nat Protoc. 2013; 8(1):17-32.

Rudrappa T, Czymmek KJ, Paré PW, Bais HP. Root-secreted malic acid recruits beneficial soil bacteria. Plant Physiol. 2008; 148(3):1547-1556.

Gordillo F, Chávez FP, Jerez CA. Motility and chemotaxis of Pseudomonas sp. B4 towards polychlorobiphenyls and chlorobenzoates. FEMS Microbiol Ecol. 2007; 60(2):322-328.

Hamon MA, Lazazzera BA. The sporulation transcription factor Spo0A is required for biofilm development in Bacillus subtilis. Mol Microbiol. 2001; 42(5):1199-1209.

Vora SM, Joshi P, Belwalkar M, Archana G. Root exudates influence chemotaxis and colonization of diverse plant growth promoting rhizobacteria in the pigeon pea – maize intercropping system. Rhizosphere. 2021; 18:100331.

Rani R, Usmani Z, Gupta P, Chandra A, Das A, Kumar V. Effects of organochlorine pesticides on plant growth-promoting traits of phosphate-solubilizing rhizobacterium, Paenibacillus sp. IITISM08. Environ. Sci Pollut Res. 2018; 25:5668-5680.

King JE. The colorimetric determination of phosphorus. Biochem J. 1932; 26:292-297.

Aasfar A, Meftah Kadmiri I, Azaroual SE, Lemriss S, Mernissi NE, Bargaz A, Zeroual Y, Hilali A. Agronomic advantage of bacterial biological nitrogen fixation on wheat plant growth under contrasting nitrogen and phosphorus regimes. Front Plant Sci. 2024; 15:1388775.

Chrastil J. Colorimetric estimation of indole-3-acetic acid. Anal. Biochem. 72, 134–138.

Glickmann, E., Dessaux, Y., 1995. A critical examination of the specificity of the Salkowski reagent for indolic compounds produced by phytopathogenic bacteria. Appl Environ Microbiol. 1976; 61(2):793-796.

Wang L, Cui YW. Mutualistic symbiosis of fungi and nitrogen-fixing bacteria in halophilic aerobic granular sludge treating nitrogen-deficient hypersaline organic wastewater. Bioresour Technol. 2024; 394:130183.

Zhu W, Lomsadze A, Borodovsky M. Ab initio gene identification in metagenomic sequences. Nucleic Acids Res. 2010; 38(12):e132-e132.

Fu L, Niu B, Zhu Z, Wu S, Li W. CD-HIT: accelerated for clustering the next-generation sequencing data. Bioinformatics. 2012; 28(23):3150-3152.

Buchfink B, Xie C, Huson DH. Fast and sensitive protein alignment using DIAMOND. Nat Methods. 2015; 12(1):59.

Huson DH, Mitra S, Ruscheweyh HJ, Weber N, Schuster SC. Integrative analysis of environmental sequences using MEGAN4. Genome Res. 2011; 21(9):1552-1560.

Chen H, Boutros PC. VennDiagram: a package for the generation of highly-customizable Venn and Euler diagrams in R. BMC bioinformatics. 2011; 12:1-7.

**Supplementary tables**

**Table S1 Information on primers used in the qPCR assay.**

| **Gene** | **Primer orientation** | **Primer sequence (5' > 3')** |
| --- | --- | --- |
| IAA30 | forward | TCAATGGCTCCGAGTACGTT |
| IAA30 | reverse | CCATCGCCCTTTGTGCTGAA |
| ARF1 | forward | TGACGGAGAAAAGAAGCCCC |
| ARF14 | reverse | CCATGCAAGTCCTTGGCAAC |
| GH3.7 | forward | GGCTTCGTGGAGTGCCTCA |
| GH3.7 | reverse | GTAGCAGGAGACAGGCACAA |
| AO2 | forward | GGCTGTTGAGCGGTTCAATG |
| AO2 | reverse | GCACTTTGTCAAGGAGTCCG |
| NPF1.2 | forward | GAGTTCCCACCAAGAAGCTGA |
| NPF1.2 | reverse | ATCTGCCGGTCCATCGTAGC |
| NPF8.4 | forward | CGGTGTACACAGAATTCTTCCAAAA |
| NPF8.4 | reverse | CATGCTCTCCAGTTGCCAGT |
| SWEET13 | forward | GAAGGCCAAGGTCTTCACGG |
| SWEET13 | reverse | ACACGACCGATGATGCTGAG |
| PPT1 | forward | CAGTCTGCCGGTCTGAACTT |
| PPT1 | reverse | CGCGGTTCCGATAGAGTTGA |
| GPT2 | forward | CGATCATAGGTGGATGCGCT |
| GPT2 | reverse | GGACAGTTCAGGTTCTTCGGA |
| PAL | forward | ATGGCGCCGCCTCAC |
| PAL | reverse | GCTCGCCATTCCACTCCTT |
| SSII | forward | AGGATTTTCTCGGGACGGAC |
| SSII | reverse | ATTTCGCTCTGCAACGCTAC |
| FDX6 | forward | CCATCTCGCGATCCGAAGTC |
| FDX6 | reverse | GGTTTGCTGTTTGCTTGGTG |
| PSB28 | forward | CGTCCCAAGAAACCTGTCCA |
| PSB28 | reverse | TGGTCGAACTCGAAGATGGC |
| AAO | forward | CAAGGACTTCAGGGGCATCA |
| AAO | reverse | TGACAGCCAAGCTGACTCTG |
| ASMT1 | forward | CACTGCTTCGCCTACGTCAA |
| ASMT1 | reverse | TGTGGGCTCCAACAAGGAGT |
| YUC3 | forward | CACTGGTTACCACAGCAACG |
| YUC3 | reverse | ACGACGGTGACATGTTGTG |
| PHO89 | forward | CTACTACAAGGGCCACCACG |
| PHO89 | reverse | CGCTCAGCCCAACCTTTTTC |
| NPF6.2 | forward | CCGACTTCATGGGCACCTC |
| NPF6.2 | reverse | AGCTGGCGCACTGTCG |
| NPF2.11 | forward | TGGCCGCATTCTTCTTCCTT |
| NPF2.11 | reverse | AGTTGACGTTGCTCTGGAGG |
| BGLU12 | forward | GACGACTTCGAGTGGTCAAG |
| BGLU12 | reverse | CTGCAACACGTACAATGCCT |
| GLN1 | forward | AGGCTGGAGTCACATGTTCG |
| GLN1 | reverse | AGCTCCATTCCAGTCTCCCT |
| Cyp4v2 | forward | TTACGAGCTTATCCACCGGC |
| Cyp4v2 | reverse | TGCGTGTCTTTTCTCGCTACT |
| CRK6 | forward | TCCACGAGCAAATCAAGCCT |
| CRK6 | reverse | TCTGGTGGCATGTAACCTCG |
| SS1 | forward | CATGGCTGCAAAGCTGACTC |
| SS1 | reverse | TTCGAACAGGGCGTCAAACT |
| Actin | forward | ATTGTGCTCAGTGGTGGGTCA |
| Actin | reverse | CCAATCCAAACACTGTACTTCCTC |

**Table S2 Evaluation statistics of sequencing data.**

| **Sample** | **Raw Data(bp)** | **CleanData(bp)** | **Raw reads** | **Clean reads(%)** | **Q20(%)** | **Q30(%)** |
| --- | --- | --- | --- | --- | --- | --- |
| NG-1 | 9955989900 | 9917031215 | 66373266 | 66146236 (99.66%) | 9670868049 (97.52%) | 9243776968 (93.21%) |
| NG-2 | 9691529100 | 9653070415 | 64610194 | 64388184 (99.66%) | 9425120287 (97.64%) | 9029695054 (93.54%) |
| NG-3 | 9405716400 | 9368646302 | 62704776 | 62486058 (99.65%) | 9145344717 (97.62%) | 8760155411 (93.51%) |
| OG-1 | 9350086200 | 9309289699 | 62333908 | 62091306 (99.61%) | 9054441023 (97.26%) | 8632551275 (92.73%) |
| OG-2 | 10667847900 | 10624458302 | 71118986 | 70860180 (99.64%) | 10359806875 (97.51%) | 9908305298 (93.26%) |
| OG-3 | 10045141200 | 10002679123 | 66967608 | 66714368 (99.62%) | 9741249363 (97.39%) | 9298333659 (92.96%) |

**Table S3 Genes abundance involved in soil microbial P-cycling.**

| **Labels** | **NG** | **OG** | **Fold(OG/NG)** | **P-value** |
| --- | --- | --- | --- | --- |
| K02037 | 0.00021508 | 0.0002068 | 0.96149462 | 0.89500879 |
| K02036 | 0.00019017 | 0.00012586 | 0.66181699 | 0.168011729 |
| K02040 | 0.00026872 | 0.00023311 | 0.86747509 | 0.49611384 |
| K07636 | 0.00019352 | 0.00044084 | 2.2779938 | 0.042320227 |
| K01126 | 0.00027432 | 0.00028461 | 1.03750277 | 0.569134517 |
| K07657 | 4.45E-05 | 6.53E-05 | 1.46820933 | 0.55475805 |
| K01507 | 0.00012413 | 5.87E-05 | 0.47313095 | 0.276957544 |
| K05813 | 1.00E-05 | 0 | 0 | 0.243671342 |
| K02044 | 0.00014595 | 0.0001116 | 0.76469565 | 0.38232987 |
| K05814 | 2.21E-05 | 1.55E-05 | 0.70045791 | 0.591028378 |
| K07658 | 0.00050669 | 0.00029047 | 0.57327818 | 0.035491096 |
| K01113 | 0.00020255 | 0.00030013 | 1.48176172 | 0.021400883 |
| K01524 | 0.00019377 | 0.0003822 | 1.9724377 | 0.047065503 |
| K02038 | 0.00022876 | 0.00022243 | 0.9723433 | 0.777072239 |
| K02041 | 3.33E-05 | 1.47E-05 | 0.44133969 | 0.370115236 |
| K02042 | 4.00E-05 | 3.48E-05 | 0.87180334 | 0.745875312 |
| K03430 | 8.05E-06 | 3.52E-06 | 0.43796483 | 0.645296117 |
| K05815 | 1.87E-05 | 8.86E-06 | 0.47414472 | 0.469813421 |
| K00117 | 9.84E-06 | 2.42E-05 | 2.45800113 | 0.279627979 |
| K05816 | 8.51E-06 | 1.47E-05 | 1.72850145 | 0.615998825 |
| K06167 | 2.17E-06 | 4.96E-05 | 22.83231027 | 0.328319007 |

**Table S4 Genes abundance involved in soil microbial N-cycling.**

| **Labels** | **NG** | **OG** | **Fold(OG/NG)** | **P-value** |
| --- | --- | --- | --- | --- |
| K00265 | 0.00056241 | 0.00040371 | 0.71781675 | 0.022069655 |
| K00266 | 0.0002804 | 0.00019234 | 0.68594033 | 0.079375588 |
| K01915 | 0.00129135 | 0.00134549 | 1.04192423 | 0.65319949 |
| K00374 | 1.40E-05 | 1.76E-05 | 1.25721541 | 0.647346169 |
| K15371 | 0.00072192 | 0.00058778 | 0.81418294 | 0.049920628 |
| K04561 | 0.00014865 | 4.03E-05 | 0.27105286 | 0.059920994 |
| K00368 | 0.00012182 | 8.49E-05 | 0.69721454 | 0.165024108 |
| K01455 | 0.00015803 | 0.00013371 | 0.84608144 | 0.517672652 |
| K00366 | 0.00023666 | 0.00013835 | 0.58462079 | 0.171273908 |
| K00459 | 0.00033192 | 0.00019136 | 0.57653237 | 0.042426923 |
| K00926 | 5.63E-05 | 0.00014831 | 2.63655962 | 0.055256569 |
| K01501 | 7.91E-05 | 0.00015292 | 1.93406521 | 0.1161612 |
| K00363 | 1.63E-05 | 2.65E-05 | 1.62202383 | 0.407013536 |
| K00261 | 0.0001235 | 0.00019701 | 1.59527909 | 0.125215432 |
| K00284 | 5.87E-05 | 0.0001912 | 3.25749608 | 0.011615088 |
| K10946 | 2.42E-05 | 5.38E-05 | 2.2183684 | 0.190759311 |
| K10945 | 7.67E-05 | 7.64E-05 | 0.99605903 | 0.993554557 |
| K01673 | 7.51E-05 | 0.00010201 | 1.35831011 | 0.100675042 |
| K00262 | 6.56E-05 | 1.54E-05 | 0.2345468 | 0.141926331 |
| K02575 | 2.55E-05 | 5.03E-05 | 1.9705298 | 0.192033418 |
| K01743 | 2.22E-05 | 1.34E-06 | 0.06052178 | 0.139896617 |
| K00372 | 9.27E-06 | 5.91E-05 | 6.38228272 | 0.021225345 |
| K02567 | 6.29E-06 | 0 | 0 | 0.422649731 |
| K15578 | 2.81E-06 | 1.47E-05 | 5.22565345 | 0.410319023 |
| K15576 | 1.96E-05 | 3.47E-05 | 1.7701539 | 0.356921869 |
| K10944 | 8.04E-05 | 9.94E-05 | 1.23649695 | 0.714589076 |
| K00362 | 2.72E-05 | 2.23E-05 | 0.82205164 | 0.814244765 |
| K00367 | 7.70E-06 | 1.44E-05 | 1.87362747 | 0.142078549 |
| K00370 | 6.71E-06 | 2.44E-05 | 3.63088979 | 0.181387751 |
| K15577 | 1.60E-05 | 1.80E-05 | 1.12728895 | 0.890527204 |
| K00260 | 2.82E-06 | 8.37E-06 | 2.96164173 | 0.398348718 |
| K00371 | 3.28E-06 | 1.92E-05 | 5.84818396 | 0.495572261 |
| K01674 | 1.33E-05 | 7.36E-05 | 5.55127243 | 0.086749721 |
| K00264 | 2.82E-06 | 6.95E-06 | 2.45921348 | 0.625531933 |
| K19823 | 1.55E-05 | 2.13E-05 | 1.3778409 | 0.711349715 |

**Table S5 Growth promoting characterization of *Phyllobacterium sp*.**

| **Growth-promoting characterization** | **Means±SE** |
| --- | --- |
| Phosphate solubilization production (μg/mL) | 87.83±4.16 |
| PSI | 1.85±0.16 |
| IAA content (μg/mL) | 35.49±4.91 |
| Nitrogenase activity (IU/L) | 183.43±4.64 |

**Table S6 Correlation analysis of root exudate compounds with relative abundance of *Phyllobacterium* sp. B68**

| **Root exudates compounds** | **Correlation coefficient** | **P-value** |
| --- | --- | --- |
| 2-Phenylglycine | 0.583812693 | 0.22377352 |
| L-Homocystine | 0.668422027 | 0.146688432 |
| D-Asparagine | 0.608477908 | 0.1999262 |
| Cordycepin | 0.813282901 | 0.049040128 |
| Sinapinic Acid | 0.747144245 | 0.087820753 |
| Sodium ferulate | 0.671049662 | 0.144514905 |
| L-Leucyl-L-alanine | 0.836087147 | 0.038099177 |

**Table S7 Quality statistics of RNA sequencing data.**

| **Sample** | **Raw Reads** | **Clean Reads** | **CleanData(bp)** | **Q20(%)** | **Q30(%)** | **GC Content(%)** |
| --- | --- | --- | --- | --- | --- | --- |
| LCK-1 | 46512606 | 46351614 | 6943868157 | 0.9731 | 0.928 | 0.5467 |
| LCK-2 | 38215108 | 38083004 | 5704411360 | 0.973 | 0.9275 | 0.5477 |
| LCK-3 | 44707604 | 44565498 | 6676767332 | 0.9768 | 0.9358 | 0.5486 |
| L68-1 | 37464160 | 37343328 | 5545569332 | 0.9789 | 0.9606 | 0.5358 |
| L68-2 | 41554724 | 41423856 | 6169642051 | 0.9727 | 0.9383 | 0.5366 |
| L68-3 | 42037572 | 41898206 | 6234268525 | 0.9781 | 0.9576 | 0.5361 |
| RCK-1 | 45324450 | 45047808 | 6656077399 | 0.9758 | 0.9549 | 0.5446 |
| RCK-2 | 38983800 | 38768290 | 5698279775 | 0.9762 | 0.9561 | 0.5423 |
| RCK-3 | 45265632 | 44984514 | 6619849862 | 0.9737 | 0.9522 | 0.5444 |
| R68-1 | 38435266 | 38231224 | 5645277859 | 0.9762 | 0.956 | 0.5349 |
| R68-2 | 42694172 | 42437090 | 6255227974 | 0.9736 | 0.9517 | 0.5377 |
| R68-3 | 47368006 | 47141396 | 6988912543 | 0.9761 | 0.9561 | 0.5291 |

**Supplementary figure**





**Fig. S1** Effects of overgrazing (OG) on *L. chinensis* rhizosphere bacterial communities. (A) Number of genes annotated in different KEGG pathways. (B) Significant differences in mean abundances of level-2 KEGG pathways between NG and OG groups. Relative abundances of (C) N and (D) P cycle-related genes.


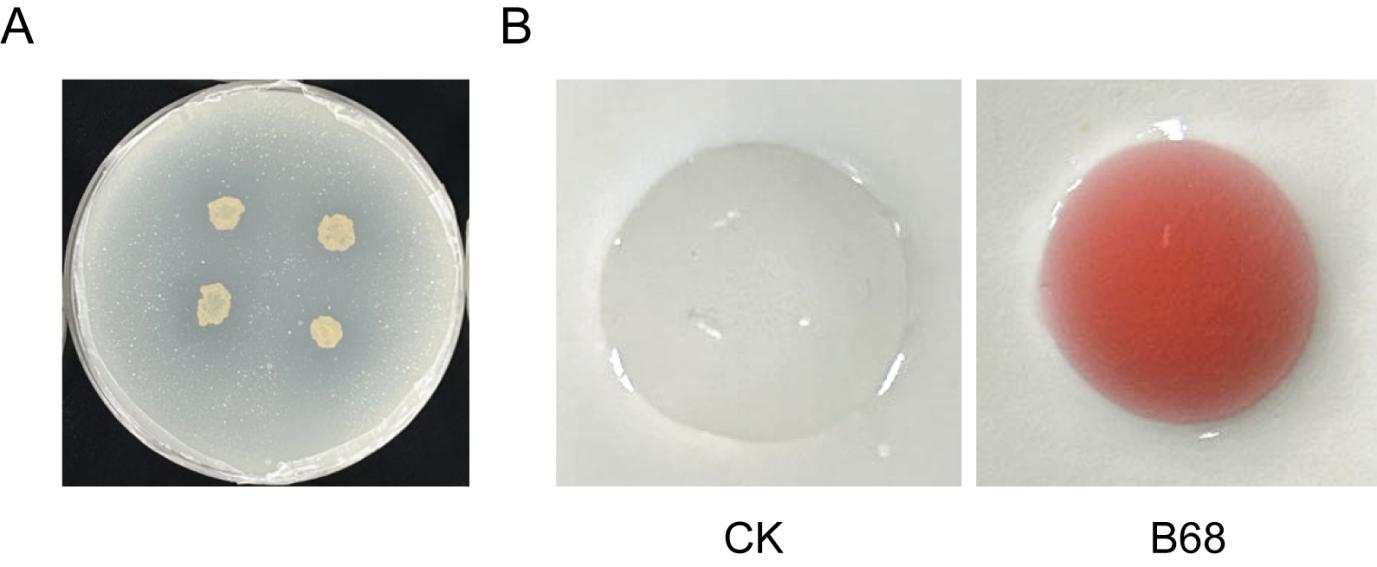


**Fig. S2** Growth-promoting properties of B68. (A) Phosphate solubilization on Pikovskaya agar of B68. Pure B68 culture was inoculated onto inorganic phosphate culture medium for qualitative analysis of phosphate solubilization. (B) IAA production of control (CK) and B68.


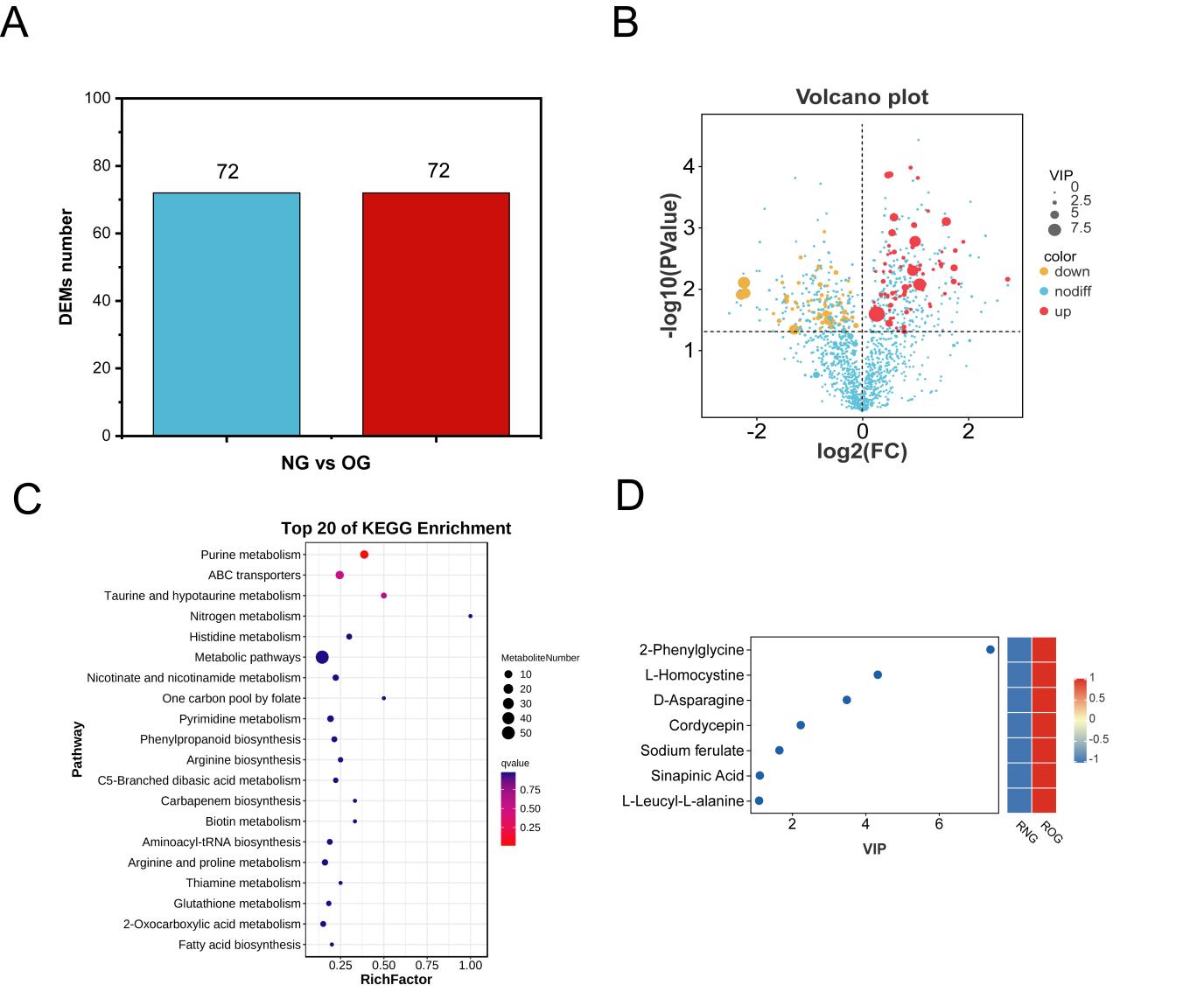


**Fig. S3** Metabolic response of *L. chinensis* root exudates to overgrazing (OG) stress. (A) Numbers of differentially expressed metabolites (DEMs) between NG and OG groups. (B) Volcano map of DEMs between NG and OG groups. VIP, variable importance in projection. (C) KEGG pathways of DEMs between NG and OG groups. (D) Variable importance in projection (VIP) values of several *L. chinensis* root exudate compounds.


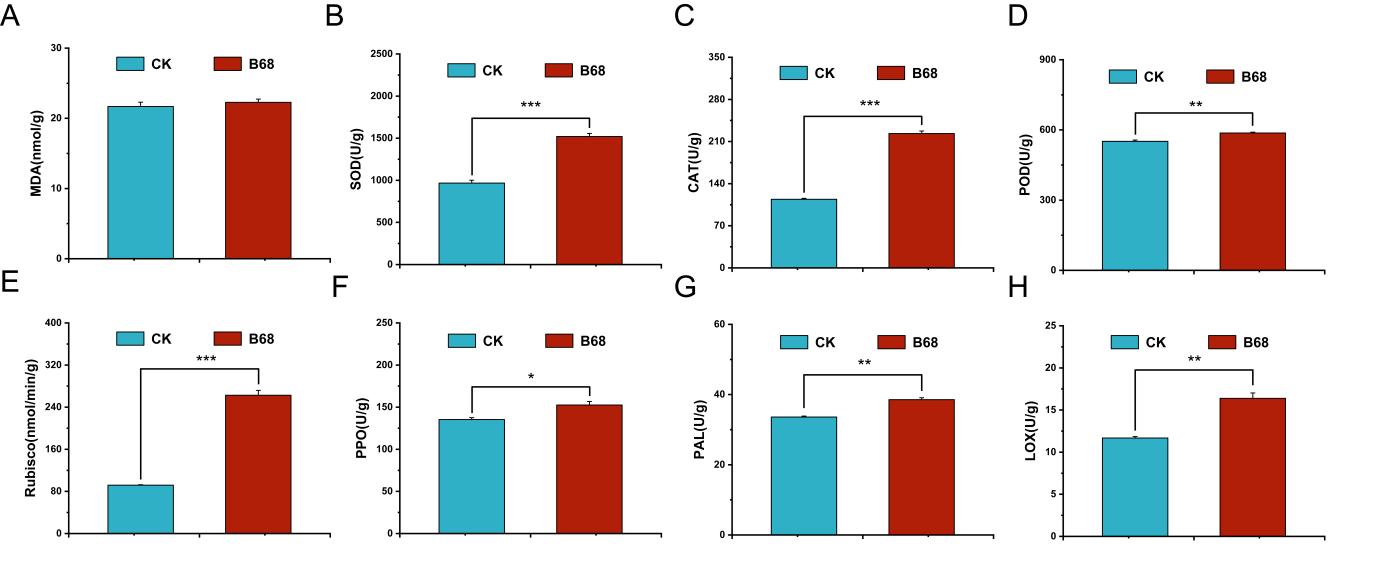


**Fig. S4** B68 inoculation affects antioxidant enzymes in *L. chinensis* leaves. (A–L) Malondialdehyde (MDA), superoxide dismutase (SOD), catalase (CAT), peroxidase (POD), RuBisCO activity, polyphenol oxidase (PPO), phenylalanine ammonia-lyase (PAL), and lipoxygenase (LOX) in leaves. CK, no inoculation; B68, inoculation with *Phyllobacterium* sp. B68. Bar chart shows mean±SE. *p<0.05; **p<0.01; ***p<0.001.


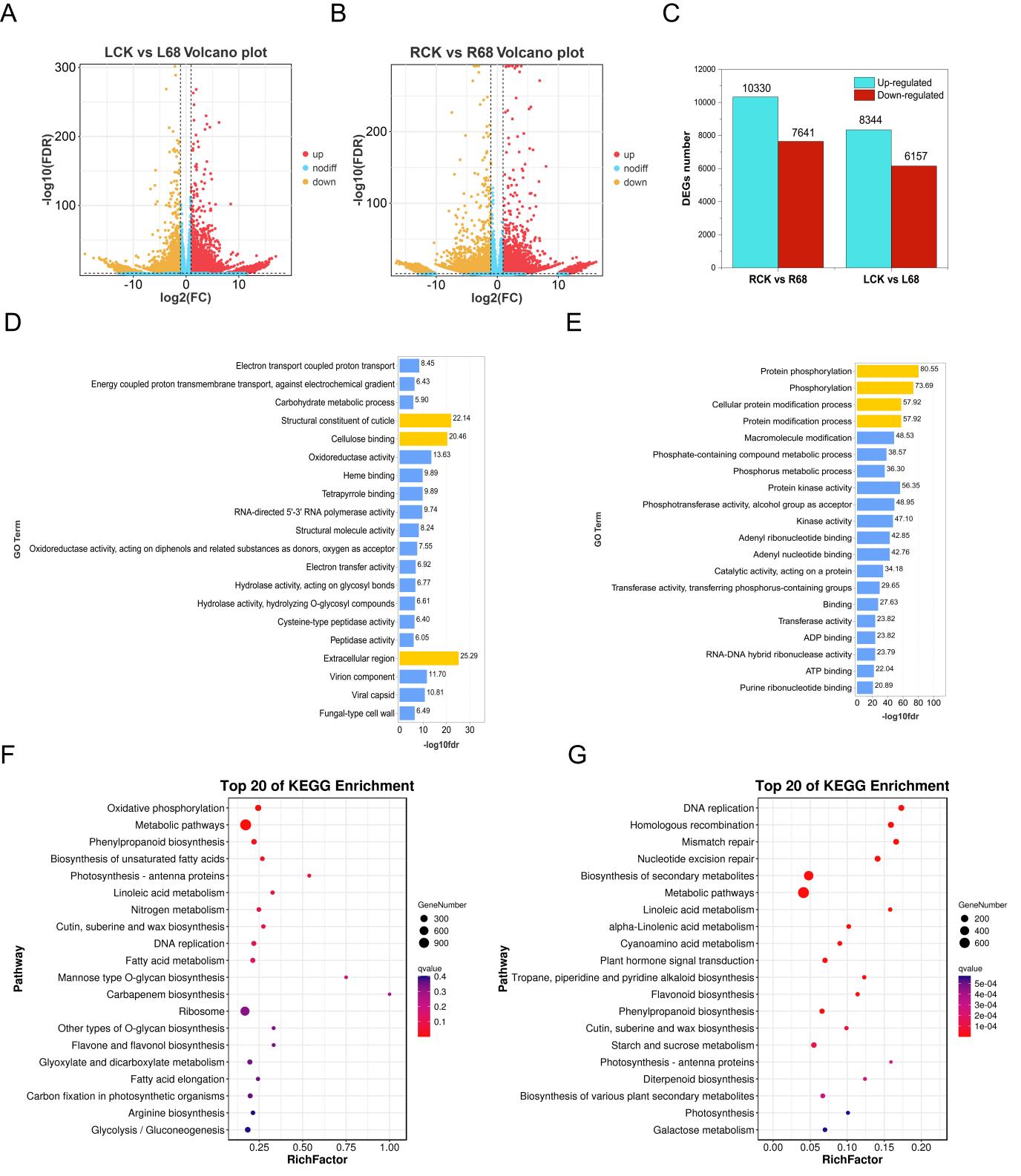


**Fig. S5** DEGs and their functions in *L. chinensis* leaves and roots. (A, B) Volcano plots of number of DEGs in the LCK vs L68 and RCK vs R68 comparisons; red, yellow, and blue circles represent the upregulated, downregulated, and non-significantly altered genes, respectively. (C) Numbers of DEGs, (D, E) KEGG analysis of DEGs, and (F, G) GO analysis of DEGs in the LCK vs L68 and RCK vs R68 comparisons. LCK, non-inoculated leaves; L68, B68-inoculated leaves; RCK, non-inoculated roots; R68, B68-inoculated roots.


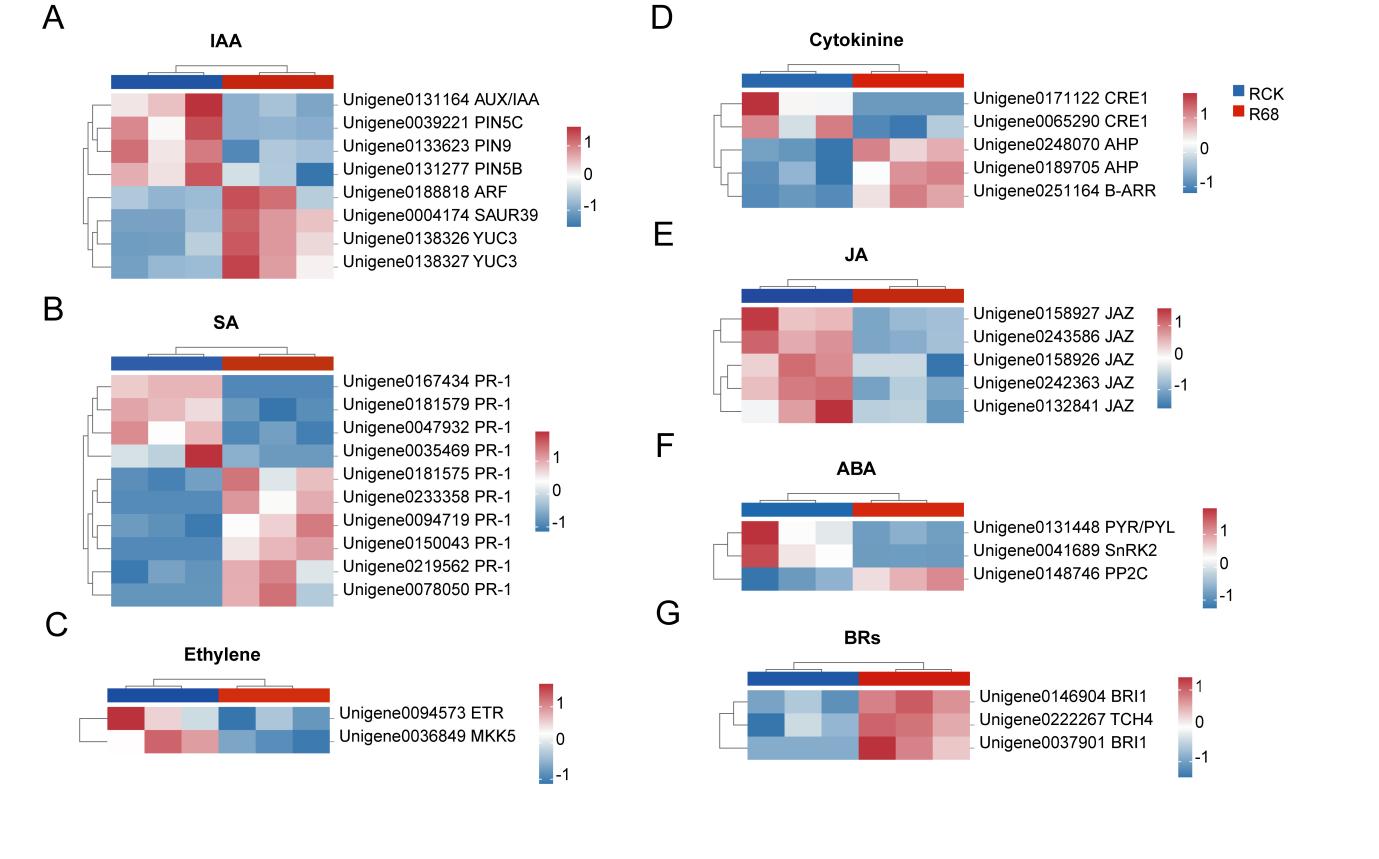


**Fig. S6** Expression of DEGs related to phytohormone signaling in roots. (A–G) Expression of DEGs related to auxin (IAA), salicylic acid (SA), ethylene (ET), cytokinin (CTK), jasmonic acid) (JA), abscisic acid (ABA), and brassinosteroid (BR) pathways in roots.

**
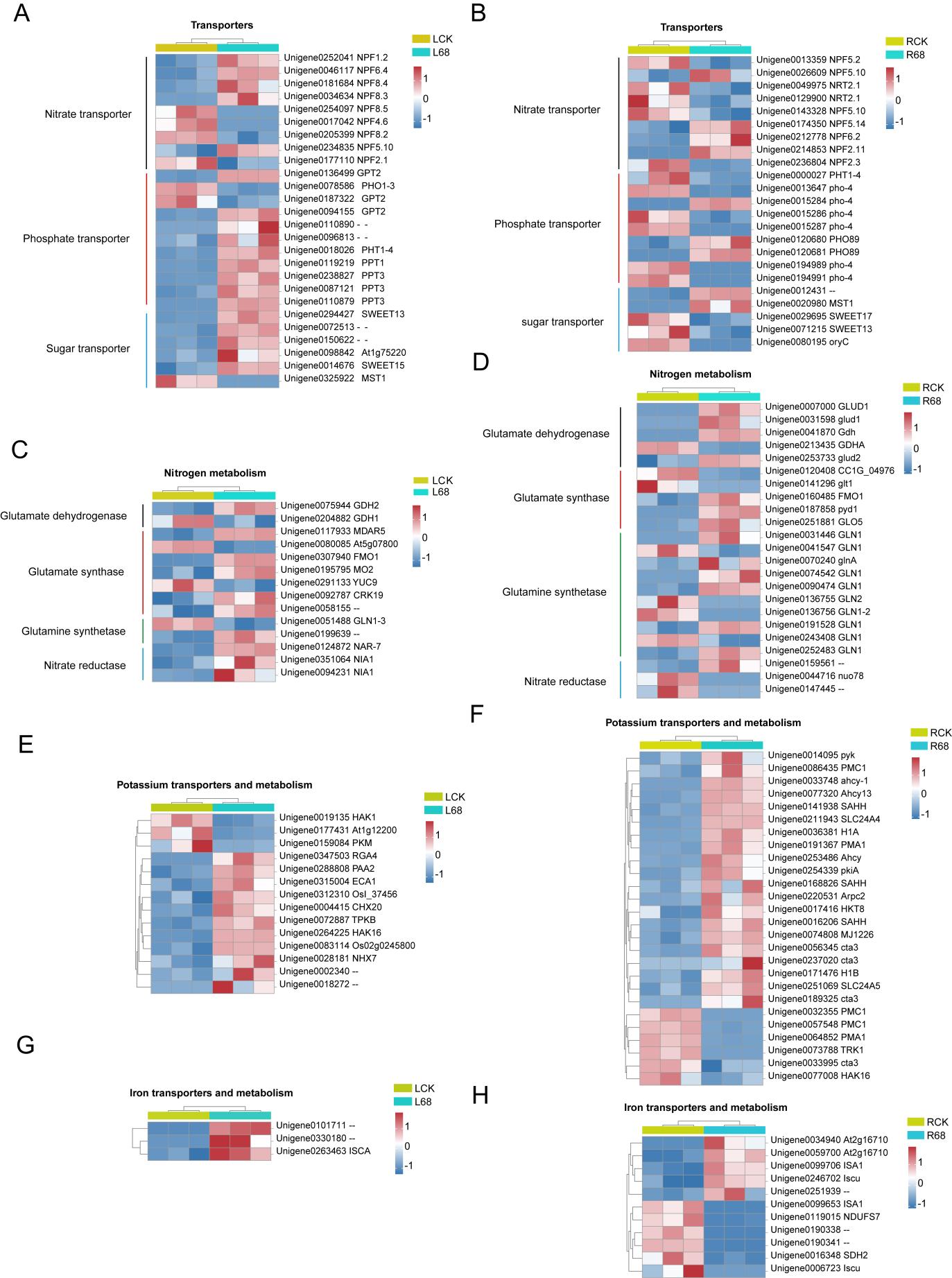
**

**Fig. S7** Expression of DEGs involved in nutrient uptake and metabolism. DEGs related to (A, B) nitrate, phosphate, and sugar transporters in leaves and roots, (C, D) N metabolism in leaves and roots, (E, F) K transporters and metabolism in leaves and roots, and (G, H) Fe transporters and metabolism in leaves and roots.


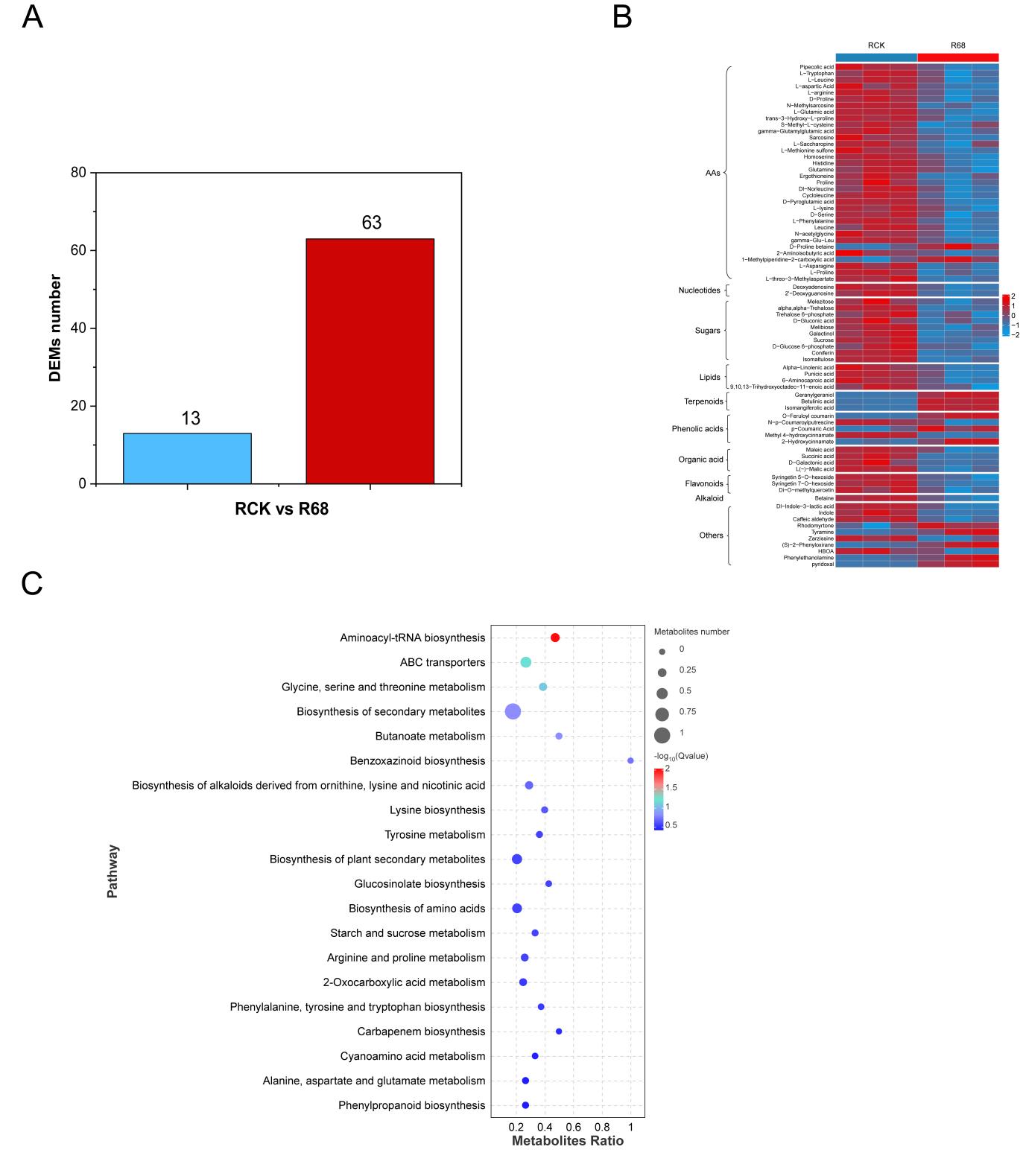


**Fig. S8** Metabolomic profiling of *L. chinensis* roots. (A) Number of differentially expressed metabolites (DEMs) between the B68-inoculated and non-inoculated groups. (B) Heatmap of changes in abundances of root DEMs. (C) KEGG analysis of DEMs.


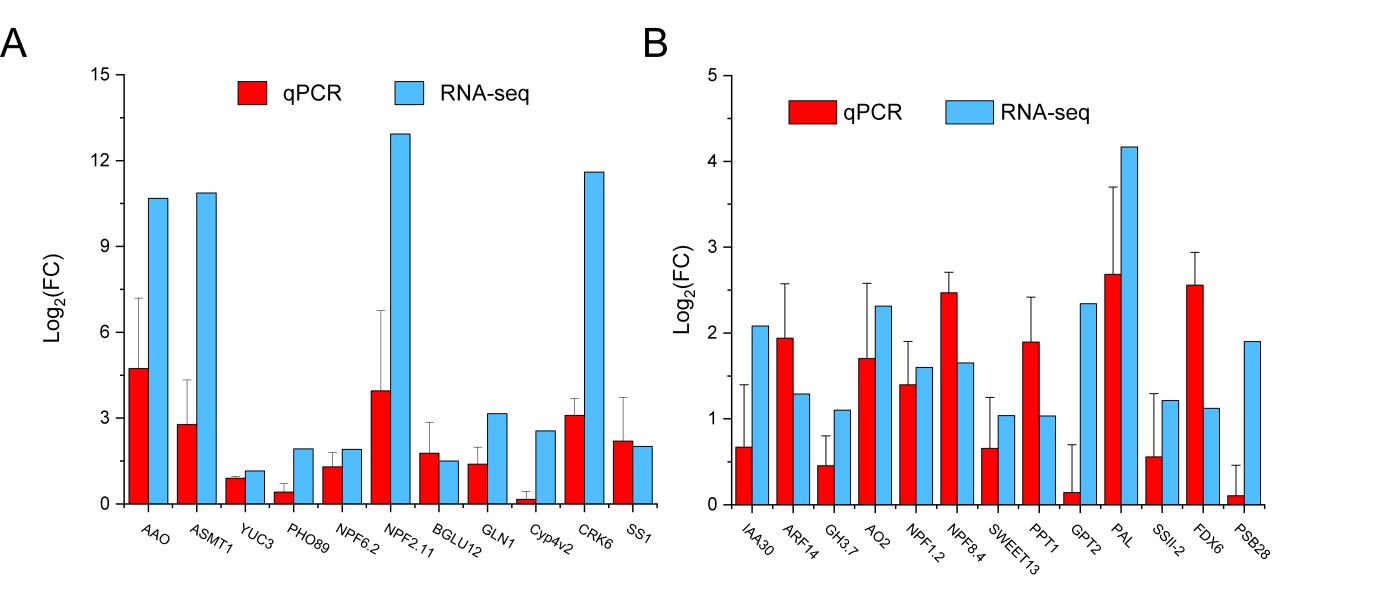


**Fig. S9** RT-qPCR validation of transcriptome data. (A) Validation of the RNA-Seq results by RT-qPCR in roots. (B) Validation of the RNA-Seq results by RT-qPCR in leaves. Data are presented as the mean ± SE of three biological replicates and two technical replicates.
